# The emerging role of ferroptosis in the pathological development and progression of sepsis

**DOI:** 10.1186/s40779-025-00665-5

**Published:** 2025-11-18

**Authors:** Hui-Ting Zhou, Jie Huang, Ya-Kun Liu, Jiang-Huai Wang, Jian Wang

**Affiliations:** 1 Institute of Pediatric Research, Children’s Hospital of Soochow University, Suzhou, 215025 Jiangsu China; 2 Department of Surgery, Children’s Hospital of Soochow University, Suzhou, 215025 Jiangsu China; 3 Department of Academic Surgery, University College Cork, Cork University Hospital, Cork, T12 DC4A Ireland

**Keywords:** Sepsis, Ferroptosis, Lipid peroxidation, Inflammation, Immune dysregulation, Organ damage, Treatment

## Abstract

Ferroptosis, a form of iron-dependent regulated cell death (RCD), is emerging as a critical mechanism in the pathogenesis and progression of sepsis. This review highlights the intricate molecular pathways and hallmark features of ferroptosis, including lipid peroxidation, dysregulation of iron metabolism, and glutathione depletion, which exacerbate sepsis progression and sepsis-associated multi-organ damage. The systemic interactions of ferroptosis with inflammation, innate, and adaptive immunity, and organ injury are elucidated, emphasizing the role ferroptosis plays both in immunity including sepsis-associated immune cell damage/dysfunction, immune dysregulation, and immunosuppression, and in sepsis-associated multi-organ injury such as acute lung injury (ALI), acute kidney injury (AKI), acute hepatic injury (AHI), acute intestinal injury, septic cardiomyopathy, and septic encephalopathy. Therapeutic strategies targeting ferroptosis hold promise for improving sepsis outcomes. Approaches include pharmacological interventions of ferroptosis-associated pathways, nanoparticle-based delivery systems, and combinatorial therapies aimed at preventing immune dysfunction and protecting against multi-organ failure. Nonetheless, challenges remain in translating preclinical findings into clinical application, necessitating further research into ferroptosis-specific regulatory networks. This review underscores the potential of therapeutics targeting ferroptosis as a transformative approach to addressing sepsis, paving the way for innovative and precision-based clinical interventions.

## Background

Sepsis, a dysregulated host response to infection, remains one of the leading causes of mortality in critically ill patients [1]. Globally, sepsis causes approximately 50 million cases and 11 million deaths annually, posing a major challenge to global health [1]. Despite the significant advancement of medical care, including fluid resuscitation and organ support, therapeutic interventions for sepsis remain palliative in nature, with no definitive treatments that directly target the underlying pathophysiological mechanisms [2]. This limitation highlights the ongoing challenge that sepsis poses to global healthcare systems, both in terms of clinical outcomes and economic burden [3]. Further research into the pathophysiology of sepsis and the development of effective drugs specifically targeting sepsis is essential. Furthermore, the underlying mechanisms of sepsis are complex and have not yet been fully elucidated [4, 5]. Traditional models of sepsis have predominantly focused on both the immune and inflammatory responses, wherein damage-associated molecular patterns (DAMPs) and pathogen-associated molecular patterns (PAMPs) activate pattern recognition receptors (PRRs), leading to a cascade of inflammatory responses that often becomes uncontrollable [6]. Critically, the dysregulation between proinflammatory responses and compensatory anti-inflammatory mechanisms can lead to profound immunosuppression, rendering patients highly susceptible to secondary infection and further complicating the pathophysiology of sepsis [4]. The complexity of these processes suggests that additional, yet to be identified, mechanisms are involved in the pathophysiology of sepsis. Further exploration of new pathways and therapeutic targets in sepsis is urgently needed.

Ferroptosis is a unique form of regulated cell death (RCD) that is distinct from apoptosis, necrosis, and autophagy [7]. The process of ferroptosis is characterized by the accumulation of iron-dependent lipid peroxides, leading to catastrophic cell membrane damage and ultimately cell death [8]. The occurrence and development of ferroptosis are driven by the failure of the cellular antioxidant defense, specifically the glutathione peroxidase 4 (GPX4) system, which normally detoxifies lipid peroxides [9]. The excessive accumulation of reactive oxygen species (ROS) and free iron plays a central role in this process, promoting an oxidative environment that exceeds the cell’s ability to maintain redox homeostasis [7, 8, 10]. Moreover, ferroptosis is inherently proinflammatory, as the release of DAMPs from the ferroptotic cell can exacerbate tissue damage [11]. This form of cell death has been implicated in a variety of diseases, including cancer, neurodegenerative disorders, and ischemia-reperfusion injury, highlighting its broad pathological relevance [8, 12]. Of note, emerging evidence suggests a close reciprocal interaction exists between the onset of ferroptosis and the development and progression of sepsis. During bacterial infection, the invading microbial pathogens require iron for their reproduction, while the host defense fights against bacterial infection by limiting the dependence of bacteria on iron, with increased iron uptake and decreased iron export to retain the iron within the cell [13, 14]. Consequently, an overload of intracellular iron occurs, which may ultimately trigger lipid peroxidation via the Fenton reaction and lead to ferroptosis [15, 16]. Once ferroptosis is initiated, the ferroptotic cells can release either excessive intracellular iron to facilitate bacterial overgrowth and aggravate the infection [14, 17] or DAMPs such as high mobility group box 1 (HMGB1) and lipid peroxides such as 4-hydroxynonenal (4-HNE) to activate inflammatory signaling pathways, intensify inflammation, and cause tissue and organ injury, thereby exacerbating the pathological progression of sepsis [18, 19]. However, the precise mechanisms by which ferroptosis contributes to the pathophysiology of sepsis remain largely unexplored, and this will therefore be an exciting frontier for future research.

In this review, we will evaluate the intricate mechanisms of ferroptosis and its distinct hallmark features during sepsis, followed by an exploration of its interplay with inflammation, immunity, and organ injury. We will also examine the impact of ferroptosis on sepsis-associated immune dysregulation, immunosuppression, and multiple organ damage, and discuss the potential of targeting ferroptosis as a new therapeutic strategy for sepsis. Through this review, we aim to provide a comprehensive understanding of ferroptosis in sepsis and highlight key areas for future research and clinical application.

## Ferroptosis in sepsis: mechanisms, hallmarks, and systemic interplay

### Immunometabolic background and therapeutic challenges in sepsis

Sepsis represents a life-threatening organ dysfunction caused by a dysregulated host response to infection, which is characterized by profound alterations in both immune and metabolic systems [20]. Despite decades of research, the pathophysiology of sepsis remains incompletely understood, and therapeutic options are still limited. The bidirectional and complex interplay between immunity and metabolism, termed immunometabolism, has emerged as a central component in the pathophysiology of sepsis and a key determinant in the clinical course and outcomes of this disease [6]. Recent advances in immunometabolism have uncovered critical insights into how metabolic reprogramming of immune cells contributes to the progression and resolution of sepsis [4, 6]. Therefore, understanding the immunometabolic background of sepsis is pivotal not only for deciphering its complex pathophysiology but also for developing effective therapeutic strategies.

#### Immunometabolic reprogramming in sepsis

Sepsis induces profound metabolic shifts across immune and parenchymal cells, which are closely intertwined with inflammatory signaling pathways. Innate immune cells such as monocytes/macrophages and neutrophils undergo rapid metabolic reprogramming upon pathogen recognition [6, 21]. During the initial hyperinflammatory phase of sepsis, monocytes/macrophages and neutrophils are not only overactivated with excessive inflammatory cytokine release but also shift their metabolic state to meet the increased energetic and biosynthetic demands of host defense [6, 21]. The hallmark of this phase is a metabolic switch from oxidative phosphorylation (OXPHOS) to aerobic glycolysis even under normoxic conditions, a phenomenon known as the Warburg effect [21]. This glycolytic reprogramming enables rapid adenosine triphosphate generation and supports inflammatory cytokine synthesis for an effective immune response. However, in the septic milieu, sustained glycolysis can become detrimental, contributing to hyperinflammation, immune dysregulation, tissue damage, and organ failure [22].

As sepsis progresses, a compensatory anti-inflammatory response often leads to immunosuppression or immunoparalysis, characterized by monocyte dysfunction, impaired antigen presentation, and T cell exhaustion [23]. Monocytes/macrophages develop immune tolerance, with reduced human leukocyte antigen-DR isotype (HLA-DR) expression, diminished inflammatory cytokine production, disrupted glycolysis, impaired mitochondrial function, excessive ROS generation, and altered fatty acid oxidation, alongside increased lymphocyte apoptosis and exhaustion [23–26]. These metabolic changes impair pathogen clearance, promote secondary infections, and drive immune dysregulation and organ failure. This biphasic immune response, initial hyperinflammation followed by immunoparalysis, is underpinned by metabolic plasticity, as immune cells enter metabolic exhaustion or tolerance after prolonged activation. Tolerized macrophages show impaired glycolysis and OXPHOS with epigenetic repression of proinflammatory genes [23, 25]. Importantly, not all immune cells respond uniformly. For instance, regulatory T cells (Tregs) sustain OXPHOS and fatty acid oxidation to promote immunosuppression, whereas effector T cells fail to maintain glycolysis, leading to functional exhaustion [23, 25]. Notably, key regulators, including AMP-activated protein kinase, hypoxia-inducible factor-1α, and mammalian target of rapamycin, as well as intermediary metabolites such as nicotinamide adenine dinucleotide (NAD⁺), succinate, and citrate, modulate this biphasic immune response via epigenetic and post-translational mechanisms [27].

#### Therapeutic challenges in sepsis

Despite advances in supportive care, targeted therapies for sepsis remain elusive [4]. Immunometabolic interventions hold promise but face significant challenges. (1) Timing and heterogeneity. The immunometabolic state of patients evolves, and therapeutic windows are narrow. Moreover, patient diversity, both genetically and with respect to the pathogen involved, complicates stratification of these patients and treatment selection [25]. One of the major challenges is temporal targeting-determining when to stimulate or suppress immunometabolic pathways during the dynamic course of sepsis. (2) The risk of metabolic modulations. Promoting glycolysis may enhance pathogen clearance early in sepsis but exacerbate tissue damage later, while enhancing OXPHOS might support immune tolerance at the cost of impaired pathogen control. Furthermore, interventions aimed at metabolic pathways such as enhancing mitochondrial function, targeting glycolytic flux, or replenishing metabolic intermediates remain under investigation. Still, they are complicated by the dual roles these pathways play in both pro- and anti-inflammatory responses [28, 29]. (3) Biomarker limitations. Despite ongoing exploration of lactate levels, cytokine profiles, and transcriptomic or metabolomic signatures to stratify patients for personalized interventions [30], there remains a lack of reliable biomarkers to accurately assess immune and metabolic states for patient stratification. Without these, tailoring immunometabolic therapies is imprecise and potentially harmful. (4) Clinical translation. Immunometabolic interventions, such as metabolic modulators, immune checkpoint inhibitors, cytokine adsorption therapies, and mitochondria-targeted therapies, have shown efficacy in preclinical models but have yet to demonstrate consistent clinical benefit and require robust clinical trial design, careful patient stratification, and real-time metabolic monitoring to bridge this translational gap [22, 31]. Moreover, emerging evidence suggests that trained immunity and epigenetic reprogramming influence long-term immune and metabolic responses to sepsis, offering potential avenues for interventions aimed at resetting maladaptive immunometabolic memory and restoring immune homeostasis [32]. Nevertheless, understanding the immunometabolic pathways involved in sepsis offers promising but complex therapeutic opportunities, while precision medicine approaches integrating dynamic immune and metabolic profiling, stratified patient cohorts, and precise intervention timing could eventually enable targeted therapies to improve outcomes of this multifaceted and deadly syndrome [33].

### Core mechanisms and key regulatory pathways of ferroptosis

**Fig. 1:**
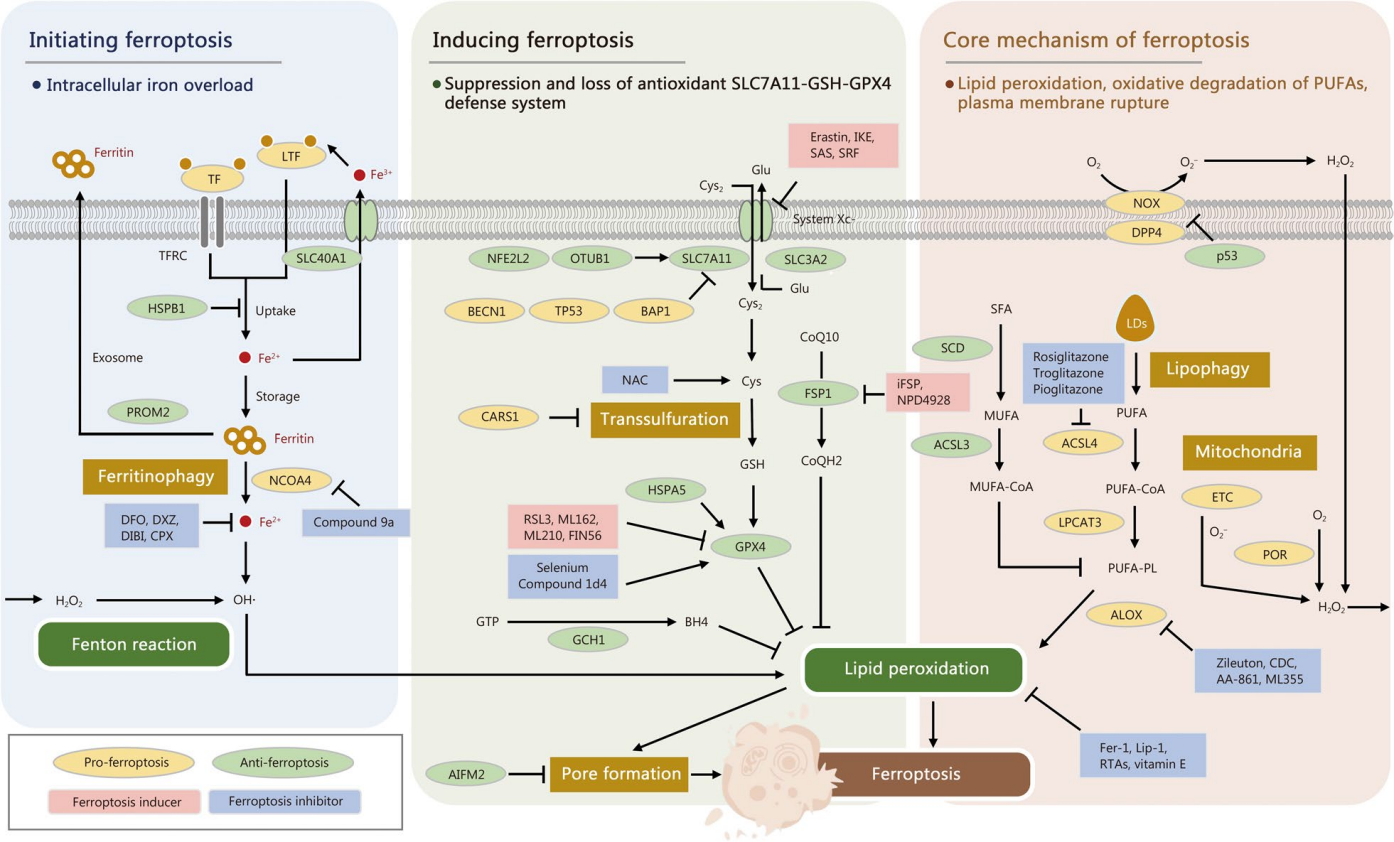
Core mechanisms, key regulatory pathways, and various inducers and inhibitors of ferroptosis during sepsis. Ferroptosis is a form of regulated cell death driven by iron-dependent lipid peroxidation. The initiation and occurrence of ferroptosis are primarily dependent on 3 major events, namely the accumulation of intracellular iron, suppression/loss of antioxidant SLC7A11-GSH-GPX4 defense system, and the generation of PUFA-enriched membrane phospholipids by ACSL4 and LPCAT3, thereby inducing lipid peroxidation. Intracellular iron overload is an important factor in initiating ferroptosis, and the ferrous iron (Fe^2+^) converts hydrogen peroxide (H_2_O_2_) into highly reactive hydroxyl radical (OH•) via the Fenton reaction to attack membrane-bound PUFA and trigger lipid peroxidation. GPX4 reduces the toxic lipid hydroperoxide to non-toxic phospholipid alcohol by converting GSH into oxidized GSH, thereby inhibiting lipid peroxidation. Both CoQH_2_ and BH4, which are regulated by either FSP1 or GCH1, also play important roles in attenuating lipid peroxidation via a GPX4-independent manner. ACSL4 and LPCAT3 are key enzymes in promoting ferroptosis, where ACSL4 esterifies PUFA into PUFA-CoA and LPCAT3 incorporates PUFA-CoA into membrane phospholipids to form PUFA-PL. Finally, ALOX catalyzes the oxidation of PUFA to drive lipid peroxidation. Consequently, lipid peroxidation directly or indirectly induces pore formation in plasma or organelle membranes, which eventually triggers ferroptotic cell death. Ferroptosis inducers and inhibitors are shown with the pink and blue backgrounds, respectively. ACSL acyl-CoA synthetase long-chain family member, AIFM2 apoptosis-inducing factor 2, ALOX arachidonate lipoxygenase, BAP1 BRCA1-associated protein-1, BECN1 beclin-1, BH4 tetrahydrobiopterin, CARS1 cysteinyl-transfer RNA synthetase 1, CDC cinnamyl-3,4-dihydroxyalpha-cyanocinnamate, CoQ10 coenzyme Q10, CoQH_2_ reduced form of coenzyme Q10, CPX ciclopirox, Cys cystine, DFO deferoxamine, DPP4 dipeptidyl peptidase 4, DXZ dexrazoxane, ETC electron transport chain, Fer-1 ferrostatin-1, FSP1 ferroptosis suppressor protein 1, GCH1 GTP cyclohydrolase 1, Glu glutamine, GPX4 glutathione peroxidase 4, GSH glutathione, GTP guanosine-5’-triphosphate, H_2_O_2_ hydrogen peroxide, HSPA5/HSPB1 heat shock protein family A member 5/family B member 1, IKE imidazole ketone erastin, LDs lipid droplets, Lip-1 lipostatin-1, *LTF* lactotransferrin, LPCAT3 lysophosphatidylcholine acyltransferase 3, MUFA monounsaturated fatty acid, MUFA-CoA monounsaturated fatty acid-Coenzyme A, NAC N-acetylcysteine, NCOA4 nuclear receptor coactivator 4, NFE2L2 nuclear factor erythroid 2-related factor 2, NOX NADPH oxidase, O_2_^*−*^ superoxide anion, OTUB1 otubain-1, POR cytochrome P450 oxidoreductase, PROM1 prominin 1, PUFA polyunsaturated fatty acid, PUFA-CoA polyunsaturated fatty acid-coenzyme A, PUFA-PL polyunsaturated fatty acid containing phospholipid, RSL3 RAS-selective lethal 3, RTAs radical-trapping antioxidants, SAS sulfasalazine, SCD stearoyl-CoA desaturase, SFA saturated fatty acid, SLC7A11/SLC40A1 solute carrier family 17 member 11/family 40 member 1, SLC3A2 solute carrier family 3A2, SRF sorafenib, TF transferrin, TFRC transferrin receptor, TP53 tumor protein p53

Ferroptosis is a unique form of RCD driven by iron-dependent lipid peroxidation and involves 3 events: intracellular iron accumulation, suppression of the solute carrier family 7 member 11 (SLC7A11)-glutathione (GSH)-GPX4 antioxidant system, and generation of polyunsaturated fatty acid (PUFA)-enriched membrane phospholipids, which promote plasma membrane lipid peroxidation and trigger cell death (Fig. 1).

#### Lipid metabolism: lipid peroxidation

Lipid peroxidation is a core mechanism of ferroptosis, characterized by the oxidative degradation of PUFAs in cell membranes [7, 12, 34]. This process is driven by ROS, especially those generated through the Fenton reaction, in which ferrous iron (Fe^2+^) catalyzes the conversion of hydrogen peroxide (H_2_O_2_) into highly reactive hydroxyl radical (OH•) [35]. These radicals initiate lipid peroxidation by abstracting hydrogen atoms from PUFAs within membrane phospholipids, leading to the formation of lipid radicals and lipid hydroperoxides (LOOH) [36, 37]. The accumulation of LOOH disrupts the integrity of the cellular membrane, ultimately causing cell death through ferroptosis [7, 38]. A key determinant of lipid peroxidation in ferroptosis is the composition of the cellular membrane, particularly the presence of PUFAs, which are highly susceptible to autoxidation, a free radical chain reaction that converts lipids into LOOH [34].

The acyl-CoA synthetase long-chain family member 4 (ACSL4) and lysophosphatidylcholine acyltransferase 3 (LPCAT3) are key enzymes in ferroptosis [39–41]. ACSL4 activates PUFAs by converting PUFAs into acyl-CoA derivatives, which are then incorporated into membrane phospholipids by LPCAT3. When the antioxidant defense system fails, these PUFA-enriched membrane phospholipids, particularly phosphatidylethanolamines (PEs), become major targets for lipid peroxidation [8]. The critical role of ACSL4 and LPCAT3 in ferroptosis lies not only in their ability to activate and integrate PUFAs, but also in their regulation of membrane phospholipid composition, directly affecting cellular sensitivity to ferroptosis [39, 40]. Previous work has shown that cells deficient in ACSL4 or LPCAT3 exhibit significant resistance to ferroptosis, highlighting the important role of these enzymes in promoting the accumulation of peroxidizable lipids in the cellular membrane [39, 40, 42, 43]. Notably, not all PUFA-containing membrane lipids exhibit the same sensitivity to ferroptosis; for instance, PEs containing arachidonic acid or adrenic acid are more susceptible to peroxidation and are closely associated with ferroptosis susceptibility [42]. In addition, certain phospholipids, such as PUFA-containing ether lipids and di-PUFA phospholipids, are also strongly associated with ferroptosis sensitivity. These findings suggest that different types of membrane phospholipids play highly specific roles in the regulation of ferroptosis.

#### Iron metabolism: intracellular iron overload

Iron is an essential element for various physiological processes, including DNA synthesis, energy metabolism, and redox reactions, and plays a critical role in various cellular functions such as the synthesis of iron-sulfur clusters, oxygen transport, and the operation of the mitochondrial respiratory chain [44, 45]. Cells transport iron from the extracellular space to the intracellular space through the transferrin receptor 1 (TFR1). Once transferrin-bound ferric iron (Fe^3+^) enters the cell, it is first incorporated into the endosome through endocytosis [44]. In the endosome, the six-transmembrane epithelial antigen prostate 3 (STEAP3) metalloreductase reduces Fe^3+^ to Fe^2+^, which is subsequently transported into the cytoplasm by the natural resistance-associated macrophage protein 2 (NRAMP2) [46, 47]. In the cytoplasm, the fate of Fe^2+^ mainly includes being oxidized to Fe^3+^ for storage in ferritin or being utilized via biochemical reactions. The nuclear receptor coactivator 4 (NCOA4)-mediated ferritinophagy breaks down ferritin and releases iron, thereby increasing the content of free iron in the cell [48]. Iron can also be exported from the cell through the only known iron exporter, ferroportin-1 (FPN1) [49]. When intracellular iron levels are insufficient, the iron regulatory protein (IRP) system is activated to maintain intracellular iron homeostasis by regulating the translation of related genes [45]. However, dysregulation of iron metabolism, especially the accumulation of excessive iron, often triggers an increase in intracellular ROS and induces lipid peroxidation, which may ultimately lead to ferroptosis [15]. At the organelle level, lysosomes and mitochondria are important centers for iron storage and regulation. Iron in lysosomes is transported to the cytoplasm through NRAMP2, while iron accumulation in mitochondria is regulated by the mitoferrin protein family, such as solute carrier family 25 member 37 and member 28, which further regulates ferroptosis by contributing to the formation of iron-sulfur clusters within cells [47, 50, 51]. In addition, the iron regulatory factors IRP1 and IRP2 control the expression of iron transport proteins such as TFR1 and FPN1 by binding to iron-responsive elements, thereby regulating intracellular iron balance. During iron overload, the IRP system becomes dysfunctional, further exacerbating iron accumulation and ferroptosis [45, 52].

#### GSH metabolism: depletion of GSH

GSH is an important antioxidant that is synthesized primarily in the cytoplasm of mammalian cells. GSH synthesis is a 2-step ATP-dependent enzymatic process involving glutamate, cysteine, and glycine [53]. The first step is that cysteine is catalyzed by glutamate-cysteine ligase to form γ-glutamylcysteine, which is the rate-limiting step in GSH synthesis and is regulated by the availability of cysteine and feedback inhibition by GSH itself [54]. The system Xc^−^ transport protein plays a crucial role in maintaining intracellular cysteine levels and promoting GSH synthesis by facilitating the exchange of extracellular cystine with intracellular glutamate [17, 53, 54]. The second step is that γ-glutamylcysteine is catalyzed by GSH synthetase, which adds glycine to γ-glutamylcysteine to generate GSH [54]. As a core component of the cellular antioxidant defense system, GSH is capable of scavenging excessive ROS and acting as an essential coenzyme for GPX4, thereby playing a key role in maintaining cellular redox balance and preventing lipid peroxide accumulation in the cell membrane and cytoplasm [17, 55]. GSH is essential for the function of GPX4, one of the most critical enzymes responsible for reducing lipid peroxides and preventing ferroptosis. When GSH levels decline, the reduction of lipid peroxides by GPX4 is impaired, leading to the accumulation of lipid peroxides and triggering ferroptosis [11, 38]. Activation of ChaC GSH-specific gamma-glutamylcyclotransferase 1, a cytoplasmic enzyme that specifically degrades GSH, can lead to GSH decline [56]. Under stress conditions such as amino acid deprivation, upregulated ChaC1 mediated by activating transcription factor 4 accelerates GSH hydrolysis, reduces its availability for GPX4, and ultimately promotes ferroptosis [57, 58]. Overexpression of multidrug resistance-associated protein 1 can excrete GSH and its conjugates from the cell, leading to decreased GSH and increasing cellular sensitivity to oxidative stress and ferroptosis [59]. Moreover, experiments that interfere with the GSH synthesis pathway demonstrate that a lack of GSH significantly enhances the sensitivity of cells to ferroptosis [56, 59]. Therefore, the interplay between GSH synthesis, degradation, and efflux plays a vital role in the regulation of ferroptosis, with declined GSH being a key factor in triggering this form of cell death.

#### GPX4-dependent regulatory pathway

The system Xc^−^-GSH-GPX4 axis is a major pathway regulating ferroptosis [15, 58]. System Xc^−^, an amino acid antiporter composed of SLC7A11 and solute carrier family 3 member 2, mediates the exchange of intracellular glutamate and extracellular cystine. Imported cystine is essential for GSH synthesis, which maintains intracellular redox balance and supports GPX4 function [18]. Various mechanisms can cause GSH depletion, including inhibition of system Xc^−^ transport proteins, a decrease in cystine uptake, and impairment in GSH synthesis [60]. Erastin, a known ferroptosis inducer, reduces intracellular GSH levels and promotes ferroptosis by inhibiting the system Xc^−^ [48, 60]. GPX4 is a selenoenzyme that plays a central role in preventing ferroptosis, and it contains selenocysteine at its active site, enabling its unique redox activity [50, 51, 61, 62]. GPX4 uses GSH as an electron donor to reduce phospholipid hydroperoxides into phospholipid alcohols, thereby preventing lipid peroxidation. The oxidized glutathione disulfide is recycled back to GSH by glutathione reductase. By directly detoxifying membrane phospholipid hydroperoxides, GPX4 serves as an irreplaceable “guardian” against ferroptosis [7, 39]. In addition, the ferroptosis inducer, RAS-selective lethal 3 (RSL3), directly targets GPX4 and inhibits its activity, resulting in the accumulation of lipid peroxides and causing cell death [9]. Notably, the loss or functional inhibition of GPX4 is often closely associated with developmental defects or organ damage. The whole-body knockout of the *GPX4* gene causes embryonic lethality in mice, while the knockout of *GPX4* in specific tissues induces spontaneous ferroptosis and oxidative stress damage [63]. These findings underscore an important role the GPX4-dependent pathway plays in regulating ferroptosis.

#### GPX4-independent regulatory pathways

The ferroptosis suppressor protein 1 (FSP1)/coenzyme Q10 (CoQ10) pathway is broadly distributed in non-mitochondrial membranes, such as the plasma membrane, and serves as a key ferroptosis defense independent of GPX4, particularly when GPX4 is absent or inhibited [38]. FSP1, a key ferroptosis inhibitor, utilizes nicotinamide adenine dinucleotide phosphate (NADPH) as an electron donor to convert oxidized CoQ10 into its reduced form, ubiquinol (CoQH_2_) [64, 65]. As a powerful radical-trapping antioxidant (RTA), CoQH_2_ suppresses lipid peroxidation by capturing lipid peroxyl radicals, thereby halting the propagation of the peroxidation chain reaction and preventing ferroptosis. Dihydroorotate dehydrogenase (DHODH) is a key mitochondrial enzyme; moreover, it plays a critical role in protecting mitochondria from ferroptosis [66]. DHODH reduces mitochondrial CoQ10 to generate CoQH_2_, thereby preventing peroxidative damage to mitochondrial membrane lipids. This mechanism is similar to the role of FSP1 in non-mitochondrial membranes but specifically targets lipid peroxidation reactions within mitochondria to inhibit mitochondrial ferroptosis. GTP cyclohydrolase 1 (GCH1), the rate-limiting enzyme for tetrahydrobiopterin (BH4) synthesis, produces BH4, while BH4, acting as a lipid-soluble antioxidant, plays a key role in inhibiting ferroptosis [67]. Functioning as a powerful RTA, BH4 can directly neutralize free radicals in the lipid peroxidation chain reaction, thereby halting the expansion of lipid peroxidation and reducing the oxidative damage to cells [67, 68]. In addition, GCH1 strengthens the antioxidant defense capacity of the membrane by modulating the lipid composition of the cell membrane, decreasing the content of PUFA-phospholipids, reducing the sensitivity of membrane lipids to oxidative damage, and increasing the levels of reduced CoQ10 [15, 67]. These 2 mechanisms work together to support the critical role the GCH1/BH4 pathway plays in ferroptosis inhibition. The dihydrofolate reductase further enhances the antioxidant capacity of the GCH1/BH4 pathway by reducing dihydrobiopterin (BH2) to BH4 [69]. Together, these GPX4-independent pathways establish a multifaceted defense system against ferroptosis by maintaining redox balance and preventing lipid peroxidation in distinct cellular compartments.

#### Other regulatory pathways

Interleukin (IL)-4 induced-1 (IL4i1) is an amino acid oxidase that inhibits ferroptosis by generating the metabolite indole-3-pyruvate. IL4i1 was originally discovered in B cells and expressed in response to IL-4 stimulation [70]. Indole-3-pyruvate suppresses ferroptosis through 2 mechanisms: 1) inhibiting the lipid peroxidation chain reaction by scavenging free radical intermediates in the lipid peroxidation process; and 2) promoting the expression of antioxidant genes such as *SLC7A11* and heme oxygenase-1 (*HO-1*), thereby alleviating oxidative damage associated with ferroptosis [71]. FSP1 participates in an atypical vitamin K cycle by reducing vitamin K to its active form, vitamin KH2, which acts as a potent free radical scavenger and lipid peroxidation inhibitor, thereby protecting cells from lipid peroxidation and ferroptosis [72].

#### The crosstalk of ferroptosis with other RCD forms

Recent studies have uncovered important crosstalk between ferroptosis and other forms of RCD, particularly pyroptosis and necroptosis [73–75]. Notably, *GPX4* deficiency in specific immune cell contexts has been shown to trigger pyroptotic cell death through NLR family pyrin domain-containing 3 (NLRP3) inflammasome activation, rather than ferroptosis, suggesting a context-dependent regulatory switch [73]. Furthermore, combined elevation of pyroptosis- and ferroptosis-associated biomarkers, such as ACSL4 and gasdermin D, has been observed in critically ill septic patients and is strongly associated with higher 28-day mortality, indicating a synergistic contribution of these pathways to immunopathology and organ injury [76]. Mechanistically, lipid peroxidation products derived from ferroptosis, such as 4-HNE and oxidized phospholipids, may further amplify pyroptosis signaling via Toll-like receptor 4 or NLRP3 activation [36, 73]. These findings highlight the need for integrated analysis of cell death networks in sepsis and open avenues for dual-pathway therapeutic targeting.

#### Sepsis-specific regulatory networks in ferroptosis

Growing evidence points to competing endogenous RNA (ceRNA) networks as pivotal in modulating ferroptosis during sepsis, where long non-coding RNAs (lncRNAs) or circular RNAs act as miRNA sponges to derepress ferroptosis effector genes [77–79]. A well-characterized axis is the lncRNA nuclear enriched abundant transcript 1 (NEAT1)-miR-9-5p-transferrin receptor (TFRC)/glutamic-oxaloacetic transaminase 1 (GOT1) axis in sepsis-associated encephalopathy (SA-E), where sepsis triggers elevated expression of exosome-derived NEAT1 in serum, which competitively binds miR-9-5p, thereby relieving its inhibitory effect on TFRC and GOT1 [77]. Upregulated TFRC enhances iron influx into endothelial cells of the blood-brain barrier, fueling the Fenton reaction and lipid peroxidation, while GOT1 disrupts glutamate metabolism, further depleting GSH and impairing GPX4 activity. This cascade exacerbates blood-brain barrier permeability and neuronal ferroptosis but can be reversed by overexpression of miR-9-5p or knocking down of NEAT1*.* In septic myocardium, lncRNA lipocalin-2 (Lcn2)-204 is the most upregulated transcript in a cecal ligation and puncture (CLP)-induced mouse model; moreover, Lcn2-204 promotes iron overload and ferroptosis by increasing expression of its host gene, *Lcn2*, a mediator of extracellular iron trafficking, while silencing Lcn2-204 reduces myocardial lipid peroxidation and confers protection against cardiac injury [78]. Similarly, in sepsis-induced acute lung injury (ALI), circ-PRKCI mitigates ferroptosis in the alveolar epithelial cells by sponging miR-382-5p, thereby limiting iron accumulation, oxidative damage, and inflammatory cytokine release [79]. Bioinformatics analyses in pediatric and postoperative sepsis have also constructed complex lncRNA-miRNA-messenger RNA (mRNA) networks involving ferroptosis-related genes such as mitogen-activated protein kinase 14 (*MAPK14*), phosphatase and tensin homolog (*PTEN*), and signal transducer and activator of transcription 3 (*STAT3*), with potential implications for immune regulation [80, 81]. These findings suggest that ceRNA networks represent an organ- and context-specific layer of ferroptosis regulation in sepsis, offering novel insights for therapeutic targeting.

### Hallmarks of ferroptosis in sepsis

**Fig. 2:**
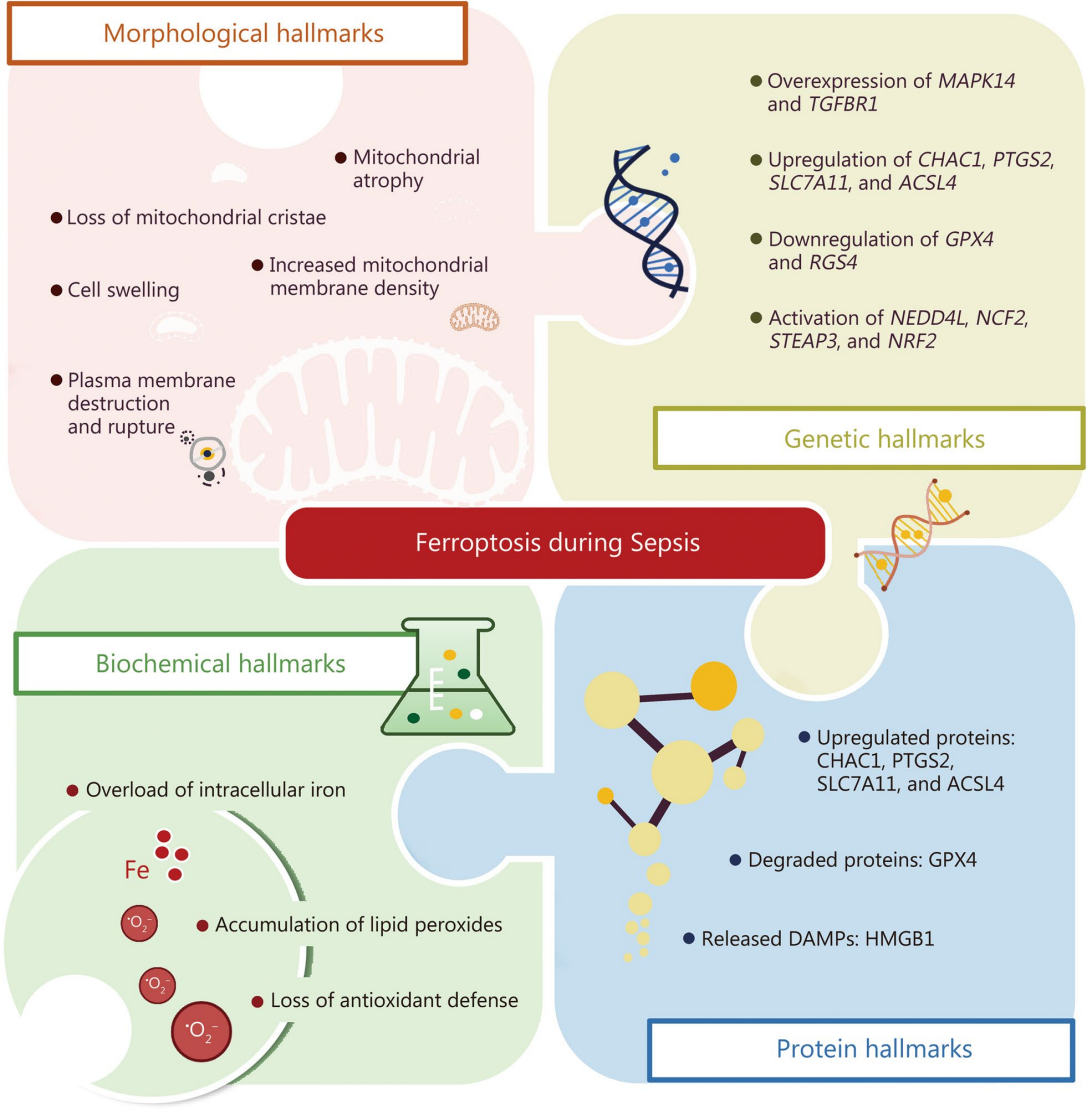
Hallmarks of ferroptosis during sepsis. Ferroptosis is driven by intracellular iron overload and lipid peroxidation and represents a major cause of cellular and tissue/organ damage during sepsis. The induction and occurrence of ferroptotic cell death are characterized by unique hallmarks with alterations in morphology, genetics, biochemistry, and proteins. The morphological hallmarks are exhibited with dramatic changes in mitochondrial ultrastructure, including mitochondrial atrophy, increased membrane density, and loss of cristae, while the biochemical hallmarks are manifested by accumulated lipid peroxides, overloaded intracellular iron, and impaired antioxidant defense system. In addition, genetic hallmarks are displayed by distinct alterations in ferroptosis-related genes and their regulatory pathways at both gene and protein levels. ACSL4 acyl-CoA synthetase long-chain family member 4, CHAC1 ChaC glutathione specific gamma-glutamylcyclotransferase 1, DAMPs damage-associated molecular patterns, GPX4 glutathione peroxidase 4, HMGB1 high mobility group box 1, MAPK14 mitogen-activated protein kinase 14, NCF2 neutrophil cytosol factor 2, NEDD4L neural precursor cell expressed developmentally downregulated gene 4-like, NRF2 nuclear factor erythroid 2-related factor 2, PTGS2 prostaglandin-endoperoxide synthase 2, RGS4 regulator of G protein signaling 4, SLC7A11 solute carrier family 17 member 11, STEAP3 six-transmembrane epithelial antigen of prostate 3, TGFBR1 transforming growth factor beta receptor 1

The induction and occurrence of ferroptotic cell death in sepsis are characterized by unique hallmarks with morphological and biochemical hallmarks, accompanied by distinct alterations at both the gene and protein levels (Fig. 2).

#### Morphological hallmarks of ferroptosis

One of the most striking morphological characteristics of ferroptosis is the dramatic alterations in mitochondrial structure, including atrophy, increased membrane density, and loss of cristae—hallmarks of ferroptosis [7, 61]. These changes result primarily from ROS generation and lipid peroxide accumulation, reflecting severe mitochondrial dysfunction. Such mitochondrial damage can be observed in multiple organs or systems affected by sepsis, including the heart, lungs, liver, and brain [10, 82]. The collapse of mitochondrial function not only leads to cell death but also exacerbates systemic organ failure as seen in severe sepsis [4, 6].

During sepsis, excessive ROS attacks membrane PUFAs, causing lipid peroxidation and accumulation of toxic byproducts such as malondialdehyde (MDA) and 4-HNE that impair membrane integrity [74]. Elevated oxidative stress amplifies this damage, leading to pore formation, increased permeability, and uncontrolled cell death [83]. Combined with mitochondrial dysfunction and iron overload, lipid peroxidation drives severe structural destruction in septic cells. In addition to changes in mitochondria and cell membranes, ferroptosis also causes damage to other organelles, such as lysosomes [84]. The disruption of lysosomal integrity, caused by ferritinophagy, exacerbates iron accumulation and further accelerates the ferroptosis process [84]. This complex interplay between iron metabolism, oxidative stress, and lipid damage constitutes a key feature of ferroptosis in sepsis and contributes to the progression of multiple organ dysfunction and failure.

#### Genetic hallmarks of ferroptosis

In recent years, research on ferroptosis has gradually revealed its underlying molecular mechanisms during sepsis, especially the related genetic markers and characteristics. Through genomics and transcriptomics analyses, scientists have confirmed that ferroptosis-related genes play a significant role in immune dysregulation and organ damage during sepsis, with studies showing that the expression of several key ferroptosis-related genes changes significantly in sepsis. For example, neural precursor cell expressed developmentally downregulated gene 4-like (*NEDD4L*) was identified from the GSE65682 dataset by multivariate survival modeling using LASSO Cox regression as part of a 14-gene ferroptosis-associated signature predictive of sepsis mortality, and it was significantly associated with patients’ survival outcomes [85]. Other studies, using differential gene expression and functional enrichment analyses, have identified a variety of ferroptosis-related genes linked to sepsis, such as neutrophil cytosol factor 2 (*NCF2*), *STEAP3* [86–88]. The upregulation of *NCF2* in sepsis-induced ALI was validated in independent mouse cohorts from Gene Expression Omnibus datasets GSE165226 and GSE168796, confirming elevated *NCF2* [86]. *NEDD4L* and *NCF4* are involved in various inflammatory and immune response regulatory pathways, such as MAPK and Janus kinase-signal transducer and activator of transcription, which not only regulate the migration and activation of immune cells but also play a critical role in the pathological process of ferroptosis [89, 90]. Despite the identification of several ferroptosis-related genes, such as *NEDD4L* and *NCF2*, through transcriptomic analyses and machine learning models, validation in independent human sepsis cohorts remains limited. In addition, ferroptosis is closely related to the miRNA network that regulates immune responses, especially mmu-miR-15a-5p, which plays a regulatory role in the process of ferroptosis by modulating *STEAP3* [46, 86].

Bioinformatics analyses have further revealed the involvement of functional enrichment and signaling pathways of ferroptosis genes in septic patients. For example, genes such as *MAPK14* and transforming growth factor beta receptor 1 *(TGFBR1)* are considered important targets for regulating ferroptosis, and these genes show significantly increased expression during multiple organ damage in septic patients [87, 88]. Downregulation of *GPX4* expression in sepsis is closely associated with the exacerbation of oxidative damage [88]. These ferroptosis-related genes and their regulatory networks provide a molecular basis for multi-organ failure in septic patients and offer potential therapeutic strategies for targeted treatment of ferroptosis [87, 88, 91]. The identification of these gene markers and signaling pathways will help further understand the role of ferroptosis in sepsis and offer new insights for future clinical research and therapeutic development.

Although direct links between ferroptosis-related genes and sepsis are limited, the GPX4 rs713041 polymorphism affects endothelial function, potentially modulating ferroptosis sensitivity in sepsis [92]. NCOA4 expression is elevated in septic patients, with higher plasma levels in non-survivors and positive correlations with sequential organ failure assessment (SOFA) scores, lactate, and proinflammatory cytokines tumor necrosis factor-α (TNF-α) and IL-6 [93]. In septic peripheral blood mononuclear cells, NCOA4 promotes ferroptosis and inflammation through the stimulator of interferon genes interaction, which can be inhibited by HET0016, a NCOA4 inhibitor [94]. Machine learning analysis of cardiac gene profiles further identifies *NCOA4* as a key ferroptosis-related gene in sepsis-induced cardiomyopathy [95].

#### Biochemical hallmarks of ferroptosis

The accumulation of lipid peroxides such as MDA and 4-HNE, generated from PUFA oxidation in cell membranes, is a hallmark of ferroptosis in sepsis. Sepsis-induced iron overload amplifies the Fenton reaction, driving PUFA peroxidation and the formation of MDA and 4-HNE, which are important markers of lipid peroxidation and are significantly elevated in septic patients [74, 96, 97]. These compounds damage the cell membrane, increasing its permeability and disrupting cellular integrity, while accumulated lipid peroxides impair cellular functions and ultimately trigger ferroptosis. Iron overload is a key feature of ferroptosis, as sepsis-induced disruptions in iron metabolism cause excessive iron accumulation and oxidative stress [14]. NCOA4-mediated ferritinophagy further releases stored iron, promoting lipid peroxidation, membrane damage, and ferroptosis; overexpression of NCOA4 increases sensitivity to ferroptosis, whereas downregulation or inhibition of NCOA4 suppresses ROS generation and ferroptotic cell death [98, 99].

Recent clinical studies have highlighted the diagnostic and prognostic potential of ferroptosis-related proteins as biomarkers for predicting the outcome of sepsis [76, 100]. In a 2025 multicenter cohort study, Zeng et al. [76] identified a panel of ferroptosis-associated proteins, including ACSL4, GPX4, and prostaglandin-endoperoxide synthase 2 (PTGS2), which were significantly correlated with sepsis severity and 28-day mortality in intensive care unit (ICU) patients. These biomarkers were shown to aid in early diagnosis, risk stratification, and differentiation of sepsis from other critical illnesses in the clinical setting, underscoring the translational and therapeutic potential of targeting ferroptosis; moreover, their elevation also correlated with multiple organ dysfunction syndrome (MODS), suggesting that ferroptosis-related proteins may serve as surrogate indicators of systemic injury burden in septic patients [76].

#### Protein hallmarks of ferroptosis

Ferroptosis during sepsis involves specific protein changes that drive lipid peroxidation and cellular damage. Key upregulated proteins include ACSL4, which facilitates the incorporation of PUFA into membrane phospholipids, thereby increasing susceptibility to oxidative damage [39, 76]. PTGS2, also known as cyclooxygenase-2, serves as a biomarker of ferroptosis, with elevated levels correlating with severity and mortality in septic patients [76]. SLC7A11, a component of the system Xc- antiporter, is upregulated to support cystine uptake for glutathione synthesis, yet its dysregulation exacerbates glutathione depletion and ferroptosis sensitivity [18, 60]. Additionally, CHAC1 promotes glutathione degradation, further impairing antioxidant defenses and accelerating lipid peroxide accumulation [56–58]. Upregulation of these proteins amplifies iron-dependent oxidative stress in septic environments.

Conversely, GPX4 undergoes degradation or functional inhibition, leading to unchecked lipid peroxidation and cell death [9, 62]. This degradation is evident in sepsis models, where reduced GPX4 correlates with organ dysfunction and inflammation [76, 100]. In addition, GPX4 has strong diagnostic and differential diagnostic value in predicting sepsis outcomes and multiple organ dysfunction [76]. Ferroptotic cells also release DAMPs like HMGB1, which activate inflammatory pathways and exacerbate tissue injury [18, 19]. This paired pattern provides a practical protein signature of ferroptosis during sepsis.

### Systemic interactions of ferroptosis with inflammation, immunity, and organ injury in sepsis

Sepsis is a life-threatening condition characterized by dysregulated host responses to infection, often causing multiple organ dysfunction. Emerging evidence implicates ferroptosis is a unique, iron-dependent form of RCD driven by lipid peroxidation in the pathogenesis and progression of sepsis. Distinct from apoptosis or necroptosis, ferroptosis is closely tied to oxidative stress, metabolic imbalance, and systemic interactions with inflammation, immunity, and organ injury.

#### The interplay between ferroptosis and inflammation

Ferroptosis is closely intertwined with the inflammatory cascade and plays a bidirectional role in modulating the inflammatory response during sepsis. The accumulation of lipid peroxides and ROS during ferroptosis acts as a proinflammatory signal to exacerbate systemic inflammation, whereas inflammation-induced dysregulation of iron metabolism and antioxidant defenses promotes the onset of ferroptosis [14, 74]. It has been shown that the accumulation of lipid peroxides and iron-mediated oxidative stress in ferroptotic cells leads to the release of DAMPs, which act as potent triggers of inflammation [8]. Among these DAMPs, HMGB1 and oxidized phospholipids activate PRRs such as Toll-like receptors and NOD-like receptors on immune cells, thereby amplifying the systemic inflammatory response [101, 102]. Once released from ferroptotic or stressed cells, HMGB1 exacerbates inflammation in sepsis, leading to inflammatory cytokine release, including IL-1β, IL-18, and tissue damage [103]. Autoantibodies against HMGB1 have proved to be associated with a favorable outcome in patients with septic shock [104]. However, this inflammatory phase is often followed by a compensatory anti-inflammatory response driven in part by prostaglandin E2 (PGE2) [105]. PGE2 acts through E-prostanoid receptor 2 and 4 receptors to suppress immune activation by inhibiting nuclear factor κB signaling and promoting the development of regulatory and immunosuppressive cell types like Tregs and M2 macrophages, while reducing the activity of dendritic cells (DCs), natural killer (NK) cells, and effector T cells [106, 107]. This shift from hyperinflammation to immunosuppression contributes to immune paralysis during the late stages of sepsis and may hinder effective pathogen clearance. Moreover, ferroptotic cell death contributes to the feedforward loop of inflammation by inducing the release of proinflammatory cytokines IL-1β, TNF-α, and IL-6. These cytokines further stimulate ROS generation and suppress the GSH-GPX4 axis by depleting intracellular GSH and downregulating GPX4 activity, thereby promoting ferroptosis [83, 101]. Additionally, inflammation drives the redistribution of iron via hepcidin-mediated degradation of ferroportin, promoting intracellular iron overload and enhancing the Fenton reaction, further accelerating lipid peroxidation and ferroptosis [108, 109]. Recent studies show that inhibiting ferroptosis with ferrostatin-1 (Fer-1) or liproxstatin-1 (Lip-1) reduces systemic inflammation and increases survival in murine models of endotoxemia and polymicrobial sepsis [110, 111], indicating that targeting ferroptosis may represent a novel anti-inflammatory therapeutic strategy in sepsis.

Clinical investigations have highlighted ferroptosis as a clinically relevant driver of sepsis inflammation. A clinical study using patient serum samples reveals reduced GPX4 expression correlating inversely with proinflammatory markers such as IL-6 and TNF-α, with GPX4 levels predicting sepsis severity in pediatric cohorts (SOFA score > 10) [100]. Similarly, elevated ACSL4 and PTGS2 in ICU sepsis patients are associated with heightened IL-6, IL-8, procalcitonin, as well as high-sensitivity C-reactive protein, demonstrating strong diagnostic performance for sepsis along with its inflammatory complications [76]. Moreover, a transcriptomic analysis integrating peripheral blood samples and machine learning approaches has identified autophagy-related protein 16-like 1 (ATG16L1) as a ferroptosis-related diagnostic marker in septic patients [112].

#### The interplay between ferroptosis and immunity

The immune response in sepsis is characterized by an initial hyperinflammatory phase followed by an immunosuppressive state. Ferroptosis contributes to both phases by modulating immune cell survival, differentiation, and functions in both innate and adaptive immunity [74, 83]. Macrophages, neutrophils, DCs, and T lymphocytes are particularly susceptible to ferroptosis under conditions of oxidative stress and iron overload common in sepsis [17]. In the early stage of sepsis, ferroptosis in innate immune cells like macrophages reduces their phagocytic and antimicrobial function but amplifies inflammation by releasing proinflammatory mediators. In the later stage of sepsis, ferroptosis depletes CD8^+^ T cells, causes T cell exhaustion and loss of cytotoxic function, and impairs antigen presentation and cytokine production in DCs, together promoting immunosuppression and increased susceptibility to secondary infections [83, 113, 114]. Activated macrophages undergo metabolic reprogramming and accumulate ROS, predisposing them to ferroptosis, particularly in iron-rich microenvironments. Additionally, ferroptosis influences the polarization of macrophages. While M1 macrophages are generally resistant to ferroptosis due to higher expression of inducible nitric oxide synthase, M2 macrophages are more vulnerable to ferroptosis, thereby altering the inflammatory milieu [115]. Notably, inhibition of ferroptosis has been shown to preserve immune cell integrity, enhance antibacterial immunity, and improve immune response in sepsis, highlighting its role in maintaining immune homeostasis [10, 116].

Transcriptomic analyses of peripheral blood from septic patients have identified hub ferroptosis-related genes (e.g., *MAPK14* and *ACSL4*) [76, 117], whose elevated expression correlates significantly with increased infiltration of activated T cells, suggesting a direct link between ferroptosis signaling and adaptive immune activation. In pediatric sepsis, MAPK3, MAPK8, peroxisome proliferator-activated receptor gamma (PPARγ), PTEN, and STAT3 similarly emerged as ferroptosis-related regulators closely associated with immune checkpoint genes and immune cell dynamics, further implicating ferroptosis in orchestrating immune responses during severe infection [81]. Postoperative sepsis data further support the role of ferroptosis-related genes such as solute carrier family 38 member 1 *(SLC38A1*), microsomal glutathione S-transferase 1 (*MGST1*), and *MAPK14* in immune dysregulation, with elevated neutrophil counts and reduced HLA-DR expression on monocytes linked to worse outcomes [80]. Thus, ferroptosis orchestrates sepsis progression by driving early hyperinflammation through innate immune cell dysfunction and later immunosuppression via T cell depletion and DC impairment, with its regulatory genes correlating with immune cell dynamics and clinical outcomes.

#### The interplay between ferroptosis and organ injury

Organ dysfunction is the hallmark of severe sepsis, with accumulating evidence implicating ferroptosis in the pathogenesis of multi-organ injury, as ferroptotic cell death contributes to the damage of key organs, including the lungs, kidneys, liver, and heart, through lipid peroxidation-mediated inflammation and tissue injury [10, 116]. In the lungs, ferroptosis exacerbates ALI by damaging alveolar epithelial cells, promoting the infiltration of inflammatory cells, impairing gas exchange, and causing barrier dysfunction [118, 119]. Ferroptosis inhibition using Lip-1 or Fer-1 alleviates pulmonary edema, attenuates inflammation, and reduces histological damage in experimental models of sepsis-induced ALI [120, 121]. Acute kidney injury (AKI) is a common complication in sepsis. Renal tubular epithelial cells are highly susceptible to ferroptosis due to their metabolic activity and iron content. Sepsis-induced oxidative stress and GSH depletion impair GPX4 activity, triggering tubular cell death and renal dysfunction, while administration of ferroptosis inhibitors demonstrates protection against tubular necrosis and preservation of renal function in murine sepsis models [121, 122]. The liver is central to metabolic regulation and detoxification in sepsis. Hepatic ferroptosis driven by mitochondrial ROS production and heme-iron metabolism contributes to hepatocyte death and liver dysfunction in sepsis, which further exacerbates systemic metabolic derangements and impairs coagulation and host defense [123, 124]. Myocardial depression is a predictor of poor prognosis in septic patients. Ferroptotic cell death of cardiomyocytes is associated with mitochondrial damage, reduced ATP synthesis, and impaired contractility, contributing to sepsis-induced cardiomyopathy [125]. The efficacy of iron chelation and lipid peroxide scavengers has been shown in improving cardiac function in preclinical models [126]. Across all these organ systems, pharmacological or genetic inhibition of ferroptosis mitigates tissue damage, reduces systemic markers of organ injury, and improves survival outcomes, highlighting ferroptosis as a unifying mechanism of organ injury in sepsis.

In a patient-level study, low circulating GPX4 and high ACSL4 independently predicted septic shock and early death, which is hypothesized to be a result of enhanced ferroptosis driven by decreased GPX4 and increased ACSL4 expression, leading to exacerbated organ dysfunction and a higher risk of early mortality [76]. In another clinical study involving septic patients, serum GSH and GPX4 levels demonstrated strong predictive value for acute gastrointestinal injury of grade II and above, suggesting that GSH and GPX4 may serve as surrogate markers of ferroptosis-driven intestinal injury and prognosis [127]. In a cohort of 50 sepsis-induced ALI patients, elevated serum ferritin and iron levels served as ferroptosis biomarkers correlating with lung tissue damage and inflammatory escalation, underscoring a potential role of iron overload in pulmonary organ injury [128]. These findings highlight the potential of targeting ferroptosis pathways as a therapeutic strategy to mitigate organ dysfunction and improve outcomes in sepsis.

## Ferroptosis in sepsis-associated immune dysregulation and immunosuppression

**Fig. 3:**
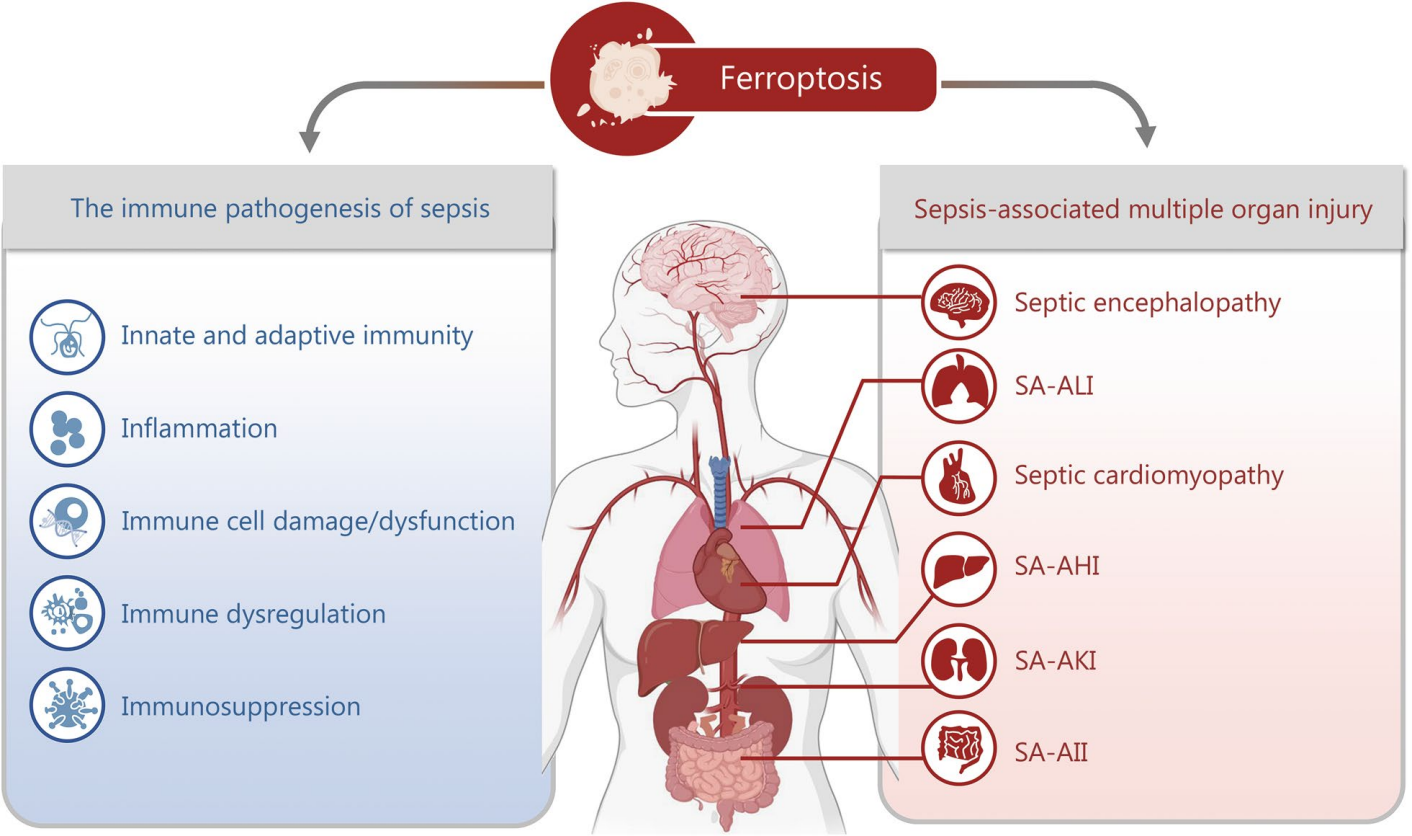
Ferroptosis in the immune pathogenesis of sepsis and sepsis-associated multiple organ injury. Ferroptosis has emerged as a pivotal mechanism in the immune pathogenesis of sepsis, contributing to sepsis-associated immune cell damage/dysfunction, immune dysregulation, and immunosuppression. Furthermore, by impairing innate and adaptive immune functions, ferroptosis influences both the hyperinflammatory phase and subsequent immunosuppressive phase during the progression of sepsis and facilitates the development of sepsis-associated multiple organ injury, including SA-ALI, SA-AKI, SA-AHI, SA-AII, septic cardiomyopathy, and septic encephalopathy. SA-AHI sepsis-associated acute hepatic injury, SA-AII sepsis-associated acute intestinal injury, SA-AKI sepsis-associated acute kidney injury, SA-ALI sepsis-associated acute lung injury

Ferroptosis has emerged as a critical player in the immune pathophysiology of sepsis, contributing to sepsis-induced immune cell damage/dysfunction, immune dysregulation, and immunosuppression. By impairing both innate and adaptive immune functions, ferroptosis accelerates the progression of sepsis from hyperinflammation to immune exhaustion and promotes the development of multiple organ injury [74, 125] (Fig. 3).

### Ferroptosis and sepsis-associated immune cell damage/dysfunction

Immune cell damage and dysfunction are central to sepsis, driving both hyperinflammation and immune paralysis. Sepsis-associated inflammation and oxidative stress promote ferroptosis, which in turn impairs the survival and function of innate and adaptive immune cells, including neutrophils, macrophages, NK cells, DCs, T cells, and B cells. This ferroptosis-induced immune dysfunction exacerbates sepsis progression and worsens disease outcomes. Macrophages are central to innate immunity and play a crucial role in sepsis through iron metabolism and inflammation. They recycle iron by engulfing senescent erythrocytes, but this makes them vulnerable to ferroptosis [129]. During sepsis, splenic red pulp macrophages increase phagocytosis, causing iron overload, lipid peroxidation, and ROS production, which promote ferroptosis [130]. Treatment with Fer-1 can reduce ROS and lipid peroxides. When exposed to the ferroptosis inducer RSL3, macrophages upregulate the iron transporter FPN1 to counteract lipid peroxidation, whereas inhibiting FPN1 raises iron levels and enhances the susceptibility of macrophages to ferroptosis [131]. Notably, different macrophage types vary in their susceptibility to ferroptosis. M1 macrophages resist ferroptosis due to nitric oxide from inducible nitric oxide synthase, which neutralizes pro-ferroptotic lipids [115]. M2 macrophages are more susceptible to ferroptosis because of impaired regulation of phospholipid oxidation, GPX4 degradation via p62/sequestosome 1 (SQSTM1)-mediated autophagy [132], higher extracellular signal-regulated kinase (ERK)-cytosolic phospholipase A2 (cPLA2)-ACSL4 activity [133], and elevated HO-1, which disrupts iron balance [134]. Sepsis-induced loss of Ras-associated protein 26 (Rab26) shifts macrophages from M1 to M2, worsening GPX4 loss and increasing ferroptosis risk [135]. Therefore, ferroptosis not only skews macrophages toward the M1 phenotype, intensifying inflammation, but also triggers HMGB1 release, recruiting more immune cells and amplifying the inflammatory response during sepsis [18]. Inhibiting ferroptosis can preserve macrophage function and reduce the inflammatory damage in sepsis [131, 136].

Neutrophils are the first line of host immune defense against infections and play an important role in sepsis through phagocytosis, degranulation, and the formation of neutrophil extracellular traps (NETs). NETs are fibrous structures composed of decondensed chromatin and antimicrobial proteins, such as elastase and myeloperoxidase, that enhance the killing of invading pathogens [137]. Studies have shown that ferroptosis is closely related to NET formation [138, 139]. Sulfasalazine, a ferroptosis inducer, promotes the generation of oxidized phospholipids in neutrophils by inhibiting SLC7A11 and arachidonate lipoxygenase, thereby accelerating the formation of NETs [138]. Moreover, ferroptosis not only affects NET formation through lipid peroxidation but also directly compromises neutrophil activity. When PUFA-phospholipids in neutrophils are oxidized, they trigger neutrophil ferroptosis and disrupt their normal function, resulting in excessive NET formation and an amplified inflammatory response [136, 140]. While this excessive inflammatory response initially aids in infection control, overwhelming neutrophil recruitment can cause tissue damage, which ultimately leads to immune dysregulation and exacerbates the progression of sepsis [136, 141]. The administration of ferroptosis inhibitors such as Fer-1 can reduce neutrophil recruitment and thus alleviate the associated tissue damage, indicating that ferroptosis is involved in promoting inflammation during sepsis [141].

Ferroptosis plays a critical role in T lymphocyte dysfunction during the progression of sepsis. A study on T cell ferroptosis found that GPX4-deficient T cells developed normally in the thymus, but peripheral CD8^+^ and CD4^+^ T cells showed impaired function. *GPX4*-knockout CD8^+^ and CD4^+^ T cells failed to expand effectively during acute infections, but this defect can be reversed with vitamin E supplementation [142]. Upregulation of GPX4 or genetic deletion of *ACSL4* also inhibited ferroptosis in CD8^+^ T cells [143]. Follicular helper T (Tfh) cells, a specialized subset of CD4^+^ T cells, play a critical role in the germinal center response. GPX4 deficiency leads to the loss of Tfh cells and germinal center responses, while selenium supplementation can increase GPX4 expression and improve antibody responses after vaccination, highlighting the importance of the selenium-GPX4-ferroptosis pathway in maintaining Tfh cell homeostasis [144]. Tregs are fundamental for maintaining immune tolerance. GPX4-deficient Tregs undergo lipid peroxidation and ferroptosis upon T cell receptor and CD28 co-stimulation, increase mitochondrial superoxide and IL-1β production, and amplify T helper 17 responses, while limiting lipid peroxidation and iron supply can reverse this process [145]. In addition, iron overload can trigger increased ROS production and DNA damage in T cells, disrupt the ratio of CD4^+^ T cells/CD8^+^ T cells, and lead to immune dysfunction [146]. Therefore, regulating T cell ferroptosis and iron metabolism may provide a new direction for improving the immune function in septic patients.

B lymphocytes are pivotal in coordinating adaptive immune responses, and damage or dysfunction in B cells can exacerbate immunosuppression during sepsis [26, 147, 148]. Marginal zone B and B1 cells, which are essential for the rapid production of immunoglobulin M antibodies, depend on GPX4 for their development and function. The highly expressed fatty acid transporter CD36 in these cells enhances fatty acid uptake, increases the intracellular levels of peroxidation-prone PUFAs, and thereby renders them highly sensitive to ferroptosis [147]. In mouse models, knockout of *GPX4* induced ferroptosis and impaired immunoglobulin M production in marginal zone B and B1 cells, leading to an impaired immune response to *Streptococcus pneumoniae* (*S. pneumoniae*) infection [147]. Furthermore, B1a cells rely more on exogenous fatty acids and autophagy-mediated lipid mobilization, which further increases intracellular free fatty acids and promotes ferroptosis [149]. Thus, ferroptosis-induced B cell dysfunction impairs antibody production, disrupts B cell homeostasis, and promotes the progression of sepsis.

### Ferroptosis and sepsis-associated immune dysregulation

Immune dysregulation in sepsis involves both exaggerated proinflammatory signaling and impaired immune surveillance [4, 25]. Ferroptosis contributes to this immune dysregulation by triggering the release of DAMPs, selectively damaging immune cell subsets, and disrupting regulatory immune cell networks [74, 102, 150]. During the early hyperinflammatory phase, ferroptosis amplifies systemic inflammation by releasing proinflammatory DAMPs such as HMGB1 and oxidized phospholipids from dying cells, and these DAMPs then activate Toll-like receptors and the NLRP3 inflammasome, perpetuating inflammatory cytokine storms [102, 151]. Additionally, lipid peroxidation during ferroptosis produces reactive aldehydes such as 4-HNE and MDA, which act as DAMPs and promote the release of proinflammatory cytokines IL-1β, TNF-α, and IL-6. Lipid ROS generated during ferroptosis also activate NLRP3, promoting IL-1β and IL-18 maturation in macrophages. Together, these processes amplify systemic inflammation and further perpetuate immune dysregulation [75, 102, 152]. Ferroptotic cell death in tissue-resident macrophages and endothelial cells promotes the recruitment of neutrophils and monocytes from the circulation. HMGB1 and oxidized phospholipids released from ferroptotic cells act as immunogenic signals that activate neutrophils and macrophages, potentially aggravating local and systemic inflammation [18, 19, 153]. This mechanism may help to explain the persistent inflammation observed in sepsis patients despite declining pathogen loads.

Conversely, ferroptosis disrupts antigen presentation and inflammatory cytokine signaling. GPX4 deficiency in DCs impairs antigen processing and T cell activation [150]. In sepsis, where DC loss and dysfunction already compromise antigen presentation, ferroptotic DC death may further weaken adaptive immunity while promoting innate immune hyperactivation and increasing susceptibility to secondary infections [114, 154]. Moreover, iron overload and lipid peroxidation in immune cells can directly suppress the expression of major histocompatibility complex (MHC) molecules and costimulatory factors necessary for T cell priming [150, 155]. Ferroptosis also weakens anti-inflammatory defenses. In Tregs, ferroptosis impairs their ability to restrain inflammation, and loss of Tregs due to ferroptosis further disrupts immune tolerance, leading to uncontrolled immune activation [145]. While the molecular pathways underlying immune dysfunction are complex, emerging evidence has implicated lipid metabolism in ferroptosis-mediated immune dysregulation. Lipidomic analyses in septic models reveal changes in phospholipid metabolism that predispose immune cells to ferroptosis and, remarkably, increased oxidation of arachidonic acid-containing phospholipids not only drives ferroptosis but also modulates immune signaling pathways, linking metabolic shifts to immune outcomes [156]. Recent work has provided compelling evidence that iron chelation with deferoxamine (DFO) improves immunologic balance and attenuates systemic inflammation in septic mice, indicating that limiting ferroptosis can modulate immune dysregulation and restore immune equilibrium [157]. Collectively, these findings highlight ferroptosis as a key contributor to immune dysregulation during sepsis.

### Ferroptosis and sepsis-associated immunosuppression

Sepsis is initially characterized by hyperinflammation, but as the disease progresses, patients often enter a state of immunosuppression. This stage is marked by lymphopenia, monocyte dysfunction with reduced HLA-DR expression, impaired pathogen clearance, and heightened susceptibility to secondary infections [148]. Ferroptosis plays a pivotal role in driving this immunosuppressive state by depleting functional immune cells, impairing their responses, and promoting immunosuppressive signals.

#### Ferroptosis in T cells and DCs, and impaired adaptive immunity

One hallmark of sepsis-induced immunosuppression is T cell exhaustion. Ferroptosis contributes to this by inducing lethal lipid peroxidation in T cells, leading to the loss of both naive and memory T cell populations [142–145]. The depletion of cytotoxic T cells compromises viral and bacterial clearance, prolonging infection, and promoting immune paralysis [158]. Mechanistically, GPX4-deficient T cells exhibit reduced proliferation and impaired cytokine production, including interferon-γ, resulting in poor pathogen clearance [159, 160]. DCs are key antigen-presenting cells essential for priming T cell responses. Ferroptosis impairs DC function by reducing MHC-II expression and costimulatory molecules such as CD80 and CD86, thereby dampening T cell activation [68, 155].

#### Monocytes, macrophages, and immunosuppressive cytokines

Monocytes and macrophages undergoing ferroptosis adopt a tolerogenic phenotype, characterized by decreased production of proinflammatory cytokines (IL-1β, TNF-α, IL-6), and increased anti-inflammatory cytokines [IL-10, transforming growth factor-β (TGF-β)], reinforcing the immunosuppressive milieu [19, 42]. Notably, M2 macrophages are more sensitive to ferroptosis than M1 macrophages, leading to a phenotypic shift from a proinflammatory M1-like state to an anti-inflammatory M2-like state. This shift further reduces HLA-DR expression and antigen presentation, weakening host defense and increasing susceptibility to secondary infections [115, 161].

Rab26, a member of the Rab GTPase family, is significantly downregulated in macrophages during the immunosuppressive phase of sepsis [135]. Loss of Rab26 not only promotes the polarization shift of macrophages from the M1 to the M2 phenotype but also accelerates GPX4 degradation, thus increasing the susceptibility of macrophages to ferroptosis. Moreover, high extracellular HMGB1 levels from damaged tissues suppress Rab26 expression, creating a vicious cycle that amplifies ferroptosis and immunosuppression [135].

#### Tregs, myeloid-derived suppressor cells (MDSCs), and selective ferroptosis resistance

Sepsis-induced immunosuppression often involves the expansion of Tregs and MDSCs. Both cell types are relatively resistant to ferroptosis due to their higher expression of antioxidant defenses, including GPX4 and system Xc^−^. Consequently, effector T cells are preferentially depleted, while suppressor cells persist, skewing the immune response toward immunosuppression [145, 162]. Moreover, ferroptotic MDSCs further suppress T cell activity and release immunosuppressive cytokines, including IL-10 and TGF-β, whereas resistant Tregs become enriched, amplifying immune paralysis [163, 164].

#### HO-1 and ferroptosis regulation in sepsis

HO-1 plays a dual role in ferroptosis during sepsis. In the early stage of sepsis, HO-1, transcriptionally activated by nuclear factor erythroid 2-related factor 2 (NRF2), exerts antioxidant and cytoprotective effects by degrading labile heme into biliverdin, carbon monoxide, and Fe^2+^, thereby alleviating oxidative stress and preserving microcirculatory integrity [83, 165]. In the late stage of sepsis, persistent HO-1 expression may elevate intracellular labile iron and ROS, ultimately triggering ferroptosis and aggravating organ damage [166, 167]. In macrophages, HO-1 modulates ferroptosis by balancing intracellular Fe^2+^ and ROS levels, while its dysregulation exacerbates oxidative stress, impairs bacterial control, and promotes immunosuppression by inducing immune cell apoptosis and shifting the cytokine profile from Th1 to Th2 [168, 169].

#### Ferroptosis-associated biomarkers and therapeutic potential

Ferroptosis-related molecules have emerged as potential biomarkers of immunosuppression in sepsis. For instance, serum GPX4 levels positively correlate with established clinical severity scores in septic patients, and alongside ACSL4, predict 28-day mortality [170]. Pharmacological inhibition of ferroptosis has shown promise in reversing immune paralysis, as administration of ferroptosis inhibitors Lip-1 or Fer-1 in murine models of late-stage sepsis demonstrates protective benefits to restore CD8^+^ T cell function, enhance bacterial clearance, and improve survival, highlighting ferroptosis as a potential therapeutic target for sepsis-induced immunosuppression [110, 116, 171].

## Ferroptosis in sepsis-associated multiple organ injury

### Ferroptosis and sepsis-associated ALI (SA-ALI)

Research in recent years has shown that ferroptosis is closely related to SA-ALI and may become a new target for the treatment of SA-ALI. In SA-ALI, excessive ROS generation and iron accumulation lead to increased lipid peroxidation in the plasma membrane, ultimately inducing ferroptosis. One study has shown that in lipopolysaccharide (LPS)-induced sepsis models, the levels of iron and ROS in alveolar epithelial cells were increased significantly, while GSH levels were decreased, and the expression of GPX4 and SLC7A11 was significantly downregulated [172]. Inhibiting ferroptosis, for example, by using the ferroptosis inhibitor Fer-1, significantly reduced sepsis-associated lung tissue damage [120]. SA-ALI can be alleviated by modulating multiple signaling pathways. For example, the NRF2 pathway inhibits ferroptosis and reduces sepsis-induced lung tissue damage by upregulating antioxidant proteins such as GPX4 and SLC7A11 [173]. The NRF2 pathway can be activated by various small molecules, such as urolithin A (UA) and uridine, which reduce lipid peroxidation and iron accumulation in the lungs, thereby protecting the lung tissues [174, 175]. In addition, NETs induce ferroptosis in alveolar epithelial cells by regulating GPX4 expression, thereby exacerbating SA-ALI [139]. Thus, ferroptosis plays a significant pathological role in SA-ALI, and modulating its related molecular mechanisms may provide potential targets for the development of new therapeutic strategies.

### Ferroptosis and sepsis-associated AKI (SA-AKI)

Ferroptosis has also been confirmed to play an important role in the pathological mechanism of SA-AKI. In the development and progression of SA-AKI, ferroptosis contributes to kidney damage through various pathways. First, iron accumulation and the oxidative stress response are the main driving factors of ferroptosis in SA-AKI [121, 176]. Iron deposition induces the generation of mitochondrial ROS, which is one of the key triggers of ferroptosis [7, 61]. Vas2870, by inhibiting NADPH oxidase, increases NADPH levels and effectively reduces the occurrence of ferroptosis in kidney cells in SA-AKI mouse models, thereby protecting kidney function [177]. Furthermore, pharmacological interventions targeting ferroptosis have shown therapeutic potential in SA-AKI. Ferroptosis inhibitors, such as Fer-1 and Lip-1, have been shown to alleviate pathological changes in SA-AKI in multiple studies [121]. In addition, several emerging small-molecule drugs, such as melatonin, have been found to inhibit sepsis-induced ferroptosis by upregulating the NRF2/HO-1 pathway, thereby improving kidney function [178]. Ginsenoside Rg1 reduced iron content in kidney tissues and renal tubular epithelial cells, while upregulating the expression of GPX4, FSP1, and GSH, and inhibiting ferroptosis by reducing iron accumulation and lipid peroxidation [179]. Further experiments have shown that the anti-ferroptosis effect of ginsenoside Rg1 depends on FSP1 protein, as its inhibitory effect on ferroptosis disappears when *FSP1* is knocked down. Moreover, it has been found that increased mitochondrial ROS not only promotes the occurrence of ferroptosis but also amplifies its damaging effects on kidney cells [180]. Marresin conjugates in tissue regeneration 1, a tissue regeneration regulator, inhibits ferroptosis by activating the NRF2 pathway and has shown significant renal protective effects in both in vivo and in vitro SA-AKI models [181]. This therapeutic strategy targeting mitochondrial ROS may provide a new treatment option for ferroptosis in SA-AKI.

One recent study has unraveled the molecular basis of ferroptosis in SA-AKI, which highlights epigenetic mechanisms in ferroptosis during SA-AKI, such as RNA m^6^A methylation modifications that enhance LPCAT3 expression, thereby exacerbating lipid peroxidation and renal tubular damage [182]. Additionally, the rhythm gene nuclear factor, interleukin 3 regulated (*NFIL3*), which is highly expressed in sepsis patients and correlates with ACSL4, modulates ferroptosis and inflammation in SA-AKI by promoting ACSL4-dependent lipid peroxidation in renal tubular epithelial cells [183]. This links circadian dysregulation and inflammation to iron-dependent cell death, while knockdown of *NFIL3* attenuates ferroptosis and inflammatory responses by downregulating ACSL4 expression, thereby protecting against SA-AKI in both in vitro and in vivo models. Furthermore, downregulation or deficiency of PRRs such as RP105 amplifies oxidative stress and ferroptosis in SA-AKI by promoting mitochondrial ROS generation, iron dysregulation, and macrophage polarization toward proinflammatory phenotypes. This, in turn, exacerbates lipid peroxidation and renal tubular damage via disruption of the HO-1/SLC7A11/GPX4 axis [184].

### Ferroptosis and sepsis-associated AHI (SA-AHI)

Sepsis leads to iron overload, decreased GSH levels, and lipid peroxide accumulation, thereby triggering hepatocyte ferroptosis and exacerbating liver injury [96]. In a murine model of CLP-induced sepsis, GSH levels in the liver were significantly reduced, while Fe^2+^ and MDA levels increased, and mitochondrial morphology was altered, manifested as mitochondrial shrinkage and increased double membrane density, the hallmarks of ferroptosis [68, 96, 123]. These changes underscore the important role of lipid peroxidation and oxidative stress in driving ferroptosis-mediated liver damage during sepsis. Targeting ferroptosis offers potential strategies to reduce liver damage. For example, the ferroptosis inhibitor Fer-1 protects the liver effectively by mitigating oxidative stress and iron overload, thereby limiting lipid peroxidation-driven hepatic tissue injury [185]. Similarly, yes-associated protein 1 (YAP1) protected the liver from sepsis-induced hepatic damage by inhibiting ferritinophagy-mediated ferroptosis, while knockout of *YAP1* aggravated liver damage in septic mice, leading to a reduction in the expression of key ferroptosis regulatory molecules such as SLC7A11 and GPX4, thereby promoting ferritin degradation and further exacerbating ferroptosis and liver damage [186]. Moreover, it has been found that the protein irisin secreted during exercise can restore mitochondrial function by regulating the ferroptosis pathway and significantly reduce liver damage in septic mice, thereby providing a new approach for the treatment of sepsis [123]. Histone modifications also play a significant role in ferroptosis susceptibility during sepsis-induced liver injury. Enhancer of zeste homolog 1, a histone modifier, promotes hepatocyte vulnerability to ferroptosis by repressing NRF2 activation, leading to excessive lipid peroxidation [187]. Conversely, the enhancer of zeste homolog 1 deficiency enhances NRF2-mediated resistance, protecting hepatocytes from oxidative damage. Additionally, Lcn2 exacerbates hepatic ferroptosis by promoting iron accumulation and oxidative stress, while its knockdown has been demonstrated to effectively alleviate liver injury via ferroptosis inhibition [188].

### Ferroptosis and sepsis-associated acute intestinal injury (SA-AII)

Ferroptosis is also involved in SA-AII. Ferroptosis contributes to intestinal mucosal damage through the generation of ROS and accumulation of lipid peroxide, thereby exacerbating intestinal ischemia-reperfusion injury [189]. In a mouse model of sepsis-induced iron overload, damage to the intestinal mucosa was associated with increased serum levels of LPS and 1,3-β-D-glucan, which further amplified the inflammatory response in sepsis [97]. In addition, research has shown that iron chelators alleviate intestinal ischemia-reperfusion injury by reducing oxidative stress and lipid peroxidation, further supporting the role of ferroptosis in SA-AII [190]. The NRF2-GPX4 signaling pathway regulates intracellular antioxidant balance, and its activation by compounds such as tert-butylhydroquinone effectively inhibits ferroptosis, alleviates intestinal damage, and offers potential therapeutic strategies for SA-AII [191].

### Ferroptosis and septic cardiomyopathy

Sepsis-associated cardiomyopathy is a common complication of sepsis, characterized by reversible left ventricular systolic dysfunction, which severely affects the prognosis of septic patients [192]. In recent years, ferroptosis, a newly discovered form of programmed cell death, has been found to play a crucial role in the pathological process of septic cardiomyopathy. Dysregulated iron metabolism and oxidative stress are considered key drivers of septic cardiomyopathy. Studies have shown that in a murine model of LPS-induced septic cardiomyopathy, ferroptosis markers in cardiomyocytes, including cyclooxygenase-2, MDA, and ROS, are significantly upregulated, accompanied by structural damage and dysfunction of mitochondria [125, 193]. These findings suggest that ferroptosis may aggravate myocardial injury by disrupting the mitochondrial function in cardiomyocytes, thereby leading to heart failure [194]. Studies have shown that inhibiting ferroptosis can reduce the onset and progression of septic cardiomyopathy. Ferroptosis inhibitors such as Fer-1 protect the heart in animal models by reducing iron accumulation and lipid peroxidation, thereby alleviating myocardial injury and improving heart function [125, 195]. Other drugs, such as dexmedetomidine (Dex), also inhibit ferroptosis-related oxidative stress by upregulating GPX4 expression and reducing iron overload in cardiomyocytes [196]. In addition, other natural compounds, such as puerarin and vitamin B6, have shown the potential to inhibit ferroptosis in septic cardiomyopathy by regulating iron metabolism and antioxidant pathways [197, 198]. These findings suggest that targeting ferroptosis may become an important strategy for the future treatment of septic cardiomyopathy.

### Ferroptosis and septic encephalopathy

SA-E is a common neurological complication in septic patients, and its pathogenesis is complex and not yet fully elucidated [199]. Recent studies have suggested that the pathological characteristics of SA-E, including neuroinflammation, oxidative stress, blood-brain barrier dysfunction, and neuronal damage, are closely linked to ferroptosis [200, 201]. In a CLP-induced sepsis model, a significant increase in ferroptosis was observed in the brain, characterized by downregulated GPX4, upregulated transferrin, and increased lipid peroxide levels [201]. In addition, ferroptosis aggravates neuronal damage by mediating glutamate excitotoxicity. This process can be reversed by the ferroptosis inhibitor Fer-1, which activates both system Xc^−^ and NRF2 and reduces the activation of the N-methyl-D-aspartate receptor subunit 2, thereby mitigating neuronal damage [202, 203]. Moreover, neuronal damage is closely related to mitochondrial dysfunction and iron overload. Studies have shown that ferroptosis not only triggers neuronal apoptosis but also affects hippocampal function, leading to cognitive impairment and memory deficits [200, 204]. By upregulating GPX4 or using ferroptosis inhibitors such as acetaminophen and Fer-1, the cognitive impairment associated with SA-E can be significantly alleviated, and the neurological function and prognosis of patients can be improved [202, 205]. Recent study has highlighted the crucial role of non-coding RNAs in regulating ferroptosis, such as the lncRNA NEAT1, a ceRNA, promotes the activation of the miR-9-5p/TFRC and GOT1 axis, further accelerating the ferroptosis process in SA-E [77].

## Targeting ferroptosis as a potential therapeutic strategy for the treatment of sepsis

**Fig. 4:**
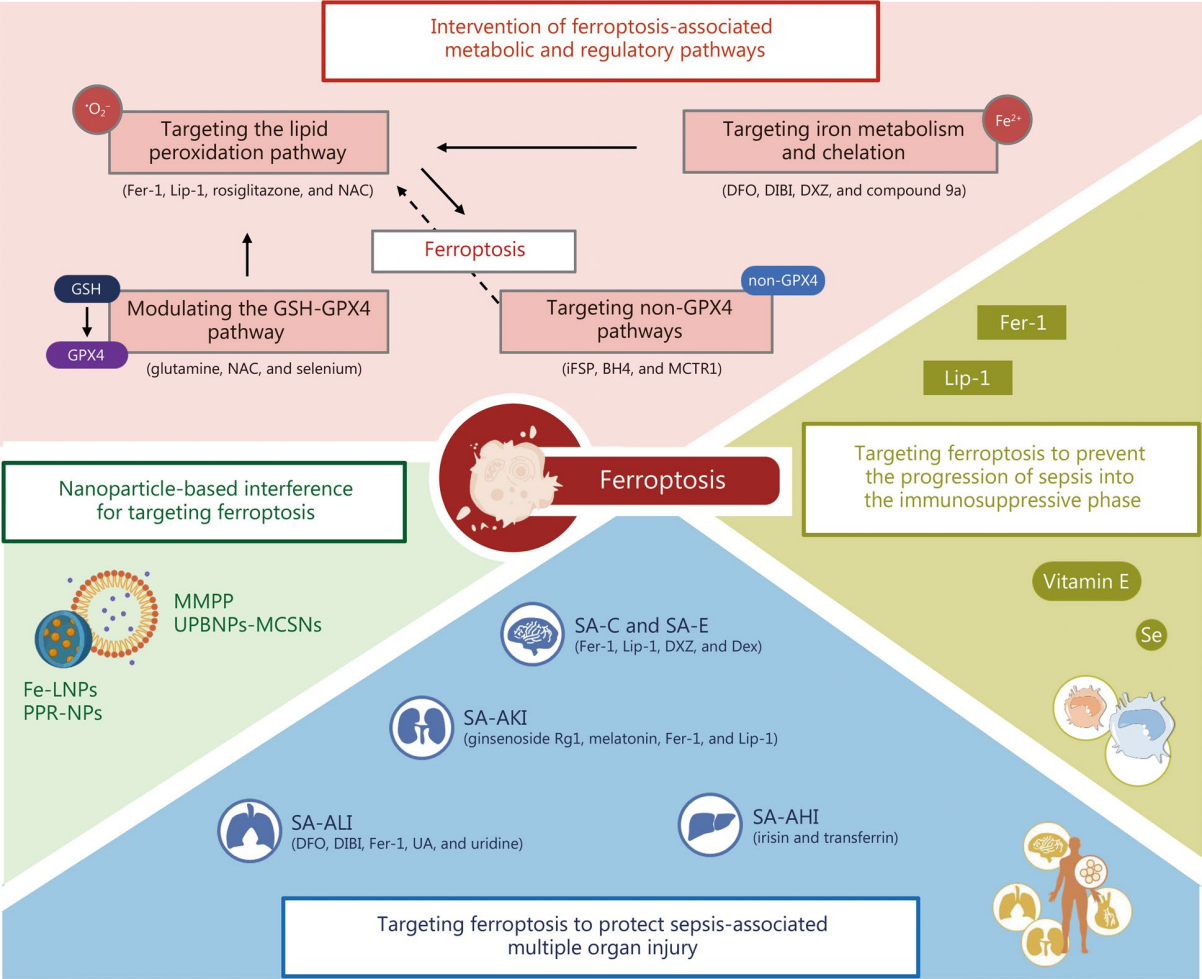
Targeting ferroptosis as a potential therapeutic strategy for the treatment of sepsis. Ferroptosis can be targeted through different approaches: 1) pharmacological intervention of ferroptosis-associated metabolic and regulatory pathways by targeting the lipid peroxidation pathway, iron metabolism and chelation, GSH-GPX4 pathway, and non-GPX4 pathways; 2) nanoparticle-based interference as a novel tool for targeting ferroptosis; 3) targeting ferroptosis to prevent the transition of sepsis from the hyperinflammatory phase to the immunosuppressive phase; and 4) targeting ferroptosis to protect sepsis-associated multiple organ damage, including SA-C, SA-E, SA-AKI, SA-ALI, and SA-AHI. BH4 tetrahydrobiopterin, Dex dexmedetomidine, DFO deferoxamine, DXZ dexrazoxane, Fe-LNPs iron-loaded lipid nanoparticles, *Fer-1* ferrostatin-1, GPX4 glutathione peroxidase 4, GSH glutathione, MCTR1 maresin conjugates in tissue regeneration 1, Lip-1 liproxstatin-1, MMPP melanin nanoparticles, NAC N-acetylcysteine, PPR-NPs polydopamine nanoparticles loaded with rutin, SA-AHI sepsis-associated acute hepatic injury, SA-AKI sepsis-associated acute kidney injury, SA-ALI sepsis-associated acute lung injury, SA-C sepsis-associated cardiomyopathy, SA-E sepsis-associated encephalopathy, Se selenium, UA urolithin A, UPBNPs-MCSNs mesoporous calcium silicate nanoparticles

The initiation and occurrence of ferroptosis can be inhibited and blocked via different approaches: 1) pharmacological modulation of ferroptosis-related metabolic and regulatory pathways; 2) nanoparticle-based approaches as innovative tools to interfere with ferroptosis; 3) ferroptosis-targeted interventions to prevent sepsis progression from the hyperinflammatory to the immunosuppressive phase; and 4) ferroptosis-focused strategies to mitigate sepsis-associated multiple organ injury, as outlined in Fig. 4 and Table 1 [39, 120, 125, 128, 131, 144, 174, 175, 178, 179, 181, 195, 196, 200, 202, 206–212].

**Table 1:** Ferroptosis inhibitors with potential therapeutic effects on sepsis

Compound name	Target	Mechanism of action	Therapeutic effects on sepsis	Efficacy metrics	References
Compound 9a	NCOA4	Disrupting the NCOA4-FTH1 interaction	Ameliorating I/R injury	IC_50_: 3.26 μmol/L (for NCOA4-FTH1 interaction) Survival rates: NR	[211]
Dex	HO-1	Activating HO-1-GPX4 pathway	Alleviating SA-C	IC_50_: NR Survival rates: improving survival from 60 to 86%	[196]
DFO	Iron	Inhibiting Fenton reaction as an iron chelator	Alleviating SA-ALI and organ damage	IC_50_: NR Survival rates: 100% at 24 h in both control and DFO groups	[208, 209]
DIBI	Iron	Preventing intracellular iron overload	Ameliorating SA-ALI	IC_50_: NR Survival rates: NR	[210]
DXZ	Iron	Attenuating intracellular iron accumulation	Alleviating SA-C	IC_50_: NR Survival rates: improved, no detailed data	[125]
Fer-1	Lipid ROS	Inhibiting lipid peroxidation as an RTA	Ameliorating SA-ALI, SA-AKI, SA-C, SA-E, and immunosuppression	IC_50_: NR Survival rates: improving survival from 20 to 40% on day 5	[120, 195, 202]
Ginsenoside Rg1	FSP1	Preventing the propagation of lipid peroxidation	Alleviating SI-AKI	IC_50_: NR Survival rates: NR	[179]
Glutamine	GSH synthesis	Enhancing GPX4 activity	Ameliorating SA-ALI	IC_50_: NR Survival rates: NR	[212]
Lip-1	Lipid ROS	Inhibiting lipid peroxidation as an RTA	Ameliorating SA-AKI, SA-C, and immunosuppression	IC_50_: NR Survival rates: NR	[131]
MCTR1	NRF2	Activating NRF2-GPX4 pathway	Alleviating SA-AKI	IC_50_: NR Survival rates: improving survival from 30 to 70% on day 7	[181]
Melatonin	NRF2/HO-1	Enhancing GPX4 activity	Alleviating SI-AKI	IC_50_: NR Survival rates: NR	[178]
NAC	GSH synthesis	Enhancing GPX4 activity	Protecting against septic lethality	IC_50_: NR Survival rates: improving survival by 16.68%	[206, 207]
Irisin	System Xc^−^/GPX4	Increasing GPX4 expression	Ameliorating SA-AHI and SA-E	IC_50_: NR Survival rates: improving survival from 40 to 70% on day 7	[181, 200]
Rosiglitazone	ACSL4	Inhibiting ACSL4-mediated lipid peroxidation	N/A	IC_50_: NR Survival rates: improving Survival against acute renal failure	[39]
Selenium	GPX4	Enhancing GPX4 activity	Ameliorating immunosuppression	IC_50_: NR Survival rates: NR	[144]
UA	NRF2	Activating NRF-HO-1 pathway	Alleviating SI-ALI	IC_50_: NR Survival rates: NR	[175]
Uridine	NRF2/ACSL4	Upregulating NRF2/downregulating ACSL4	Alleviating SI-ALI	IC_50_: NR Survival rates: NR	[174]
Vitamin E	Lipid ROS	Inhibiting lipid peroxidation as an RTA	Ameliorating immunosuppression	IC_50_: NR Survival rates: NR	[128]

*ACSL4* acyl-CoA synthetase long-chain family member 4, *Dex* dexmedetomidine, *DFO* deferoxamine, *DXZ* dexrazoxane, *Fer-1* ferrostatin-1, *FSP1* ferroptosis suppressor protein 1, *FTH1* ferritin heavy chain 1, *GPX4* glutathione peroxidase 4, *GSH* glutathione, *HO-1* heme oxygenase-1, *I/R* ischemia/reperfusion, *Lip-1* lipostatin-1, *MCTR1* maresin conjugates in tissue regeneration 1, *NAC* N-acetylcysteine, *N/A* not applicable, *NCOA4* nuclear receptor coactivator 4, *NR* not reported, *NRF2* nuclear factor erythroid 2-related factor 2, *ROS* reactive oxygen species, *RTA* radical-trapping antioxidant, *SA-AHI* sepsis-associated acute hepatic injury, *SA-AKI* sepsis-associated acute kidney injury, *SA-ALI* sepsis-associated acute lung injury, *SA-C* sepsis-associated cardiomyopathy, *SA-E* sepsis-associated encephalopathy, *UA* urolithin A

### Pharmacological intervention of ferroptosis-associated metabolic and regulatory pathways

#### Interventions targeting the lipid peroxidation pathway

In sepsis, excessive lipid peroxidation exacerbates tissue damage, especially damage to organs such as the lungs. Fer-1 and Lip-1 are small-molecule inhibitors that effectively inhibit membrane lipid peroxidation [213]. As a potent free radical scavenger, Fer-1 captures oxygen free radicals to inhibit lipid peroxidation, protects the integrity of the cellular membrane, and thus halts ferroptosis. It has been demonstrated that Fer-1 exhibits a protective effect against ALI in sepsis models [120]. By inhibiting the chain reaction of lipid peroxidation, Fer-1 attenuates cell death under septic conditions and displays a protective effect against sepsis-induced myocardial injury, lung damage, and encephalopathy [120, 195, 202]. Lip-1 has a similar effect to Fer-1 but is more effective than Fer-1 in inhibiting ferroptosis in vivo [195]. Rosiglitazone, a type of thiazolidinedione drug commonly used as a PPARγ agonist for treating type 2 diabetes, has been shown to inhibit ACSL4. By reducing the activity of ACSL4, rosiglitazone limits the incorporation of PUFAs into membrane phospholipids and reduces lipid peroxidation, thereby inhibiting the occurrence of ferroptosis [39]. ROS are core factors in the initiation and propagation of lipid peroxidation in ferroptosis. Excessive ROS, through iron-catalyzed reactions such as the Fenton reaction, drives oxidative damage to lipids, proteins, and DNA. Therefore, reducing ROS levels is a key intervention to prevent ferroptosis. N-acetylcysteine, a widely used antioxidant, strongly reduces ROS levels by replenishing intracellular GSH and directly scavenging free radicals, thereby significantly protecting against sepsis-associated lethality in sepsis animal models [206, 207, 214].

#### Interventions targeting iron metabolism and chelation

The imbalance of iron metabolism is a key factor in promoting ferroptosis, especially in sepsis, where iron overload catalyzes the generation of ROS via the Fenton reaction, leading to lipid peroxidation. In a sepsis-related study, the iron chelator DFO has been shown to protect organs by reducing systemic inflammation and oxidative stress from abnormal iron metabolism, particularly in ALI and multi-organ failure [208]. In addition, iron chelators can restore the function of phagocytes and enhance the ability of the host to clear pathogens, thereby reducing sepsis-associated tissue and organ damage [209]. Some new iron chelators, such as 3-hydroxypyridin-4-one DIBI, have demonstrated significant therapeutic effects in experimental sepsis models and can effectively reduce leukocyte infiltration in the intestinal microcirculation, suggesting that iron chelators may play a vital role in future sepsis treatment [210]. Ferritinophagy is the process of releasing free iron through ferritin degradation, and elevated free iron may further exacerbate ferroptotic cell death. NCOA4-mediated ferritin degradation increases the intracellular iron pool, thereby promoting lipid peroxidation and cell death [48]. Therefore, inhibiting NCOA4-mediated ferritinophagy to prevent free iron release could be a potential therapeutic strategy for mitigating ferroptosis. A new compound named 9a has been shown to effectively inhibit ferritinophagy-induced ferroptosis by competitively binding to NCOA4 [211].

#### Modulating the GSH-GPX4 pathway

GSH depletion is one of the main drivers of ferroptosis, and the reduction of intracellular GSH levels directly affects GPX4 activity. Glutamine, as the precursor of GSH, enhances the antioxidant capacity by providing the raw materials for GSH synthesis, thereby preventing cell death in different tissues and organs during sepsis [212]. GPX4 is a key enzyme for regulating ferroptosis, and reduced GPX4 activity directly leads to a decrease in the cell’s ability to resist lipid peroxidation, ultimately triggering ferroptosis. Selenium, essential for GPX4 function, enhances the activity of GPX4 through supplementation or selenium-dependent drugs, thereby attenuating the occurrence of ferroptosis [215]. One study has shown that selenium supplementation significantly enhances the activity of GPX4 and reduces ferroptosis in immune cells under oxidative stress environments [144]. It has been noted that selenium-dependent drugs not only reduce oxidative stress-induced organ damage by maintaining the function of GPX4 but also show potential application in other oxidative stress-related diseases [216]. Thus, regulating selenium levels to activate GPX4 may be a promising strategy in sepsis treatment.

#### Interventions targeting non-GPX4 pathways

FSP1 is an important ferroptosis inhibitory enzyme parallel to GPX4. FSP1 catalyzes the reduction of CoQ10 and converts it into antioxidant CoQH_2_, thereby preventing the propagation of the chain reaction of lipid peroxidation [64]. This mechanism plays a crucial role in the protection of cell membranes, especially when the GPX4 function is impaired or absent. Thus, the FSP1/CoQ10 pathway serves as a key defense mechanism [65]. BH4 is another potential ferroptosis regulatory component that protects cell membranes by reducing lipid peroxidation and maintaining membrane integrity. Marresin conjugates in tissue regeneration 1 can inhibit ferroptosis by activating the NRF2 signaling pathway, thereby alleviating SA-AKI [181]. NRF2 can upregulate GCH1 activity, promote BH4 synthesis, and protect cells from radiation-induced oxidative damage by reducing ROS [217]. Therefore, intervening in ferroptosis by increasing BH4 levels or activating the GCH1 pathway could be a potential strategy for treating sepsis-induced tissue damage.

Despite promising preclinical and clinical evidence, its efficacy in sepsis appears context-dependent [218]. For instance, in mouse models, the ferroptosis inhibitor UAMC-3203 failed to reduce plasma injury biomarkers in TNF-induced systemic inflammatory response syndrome or CLP-induced septic shock, despite efficacy in non-septic multiorgan dysfunction like iron overload-induced MODS; moreover, overexpression of GPX4 did not enhance survival in these sepsis models [218]. This suggests that ferroptosis may not be the predominant mode of cell death in certain septic contexts, especially when systemic inflammation is the primary driver [74].

### Nanoparticle-based interference as a novel tool for targeting ferroptosis

Nanoparticles have certain advantages in the treatment of targeting ferroptosis. Their ability to cross biological barriers enhances the drug bioavailability and is ideal for delivering therapeutic agents. Nanoparticles can encapsulate ferroptosis inducers and improve the stability and precise delivery of these drugs to targeted structures [219]. Nanoparticles can also accelerate the Fenton reaction by increasing the local concentration of reactants, such as Fe^2+^, Fe^3+^, and H_2_O_2_, further enhancing the efficacy of ferroptosis-based therapies [35]. One study has shown that Fe_3_O_4_/Gd_2_O_3_ hybrid nanoparticles loaded with cisplatin can successfully cross the blood-brain barrier and deliver key Fenton reaction reactants to the tumor site, significantly inhibiting tumor growth [35]. Recent studies have further revealed the potential of nanoparticles in the treatment of sepsis-related organ damage associated with ferroptosis [220, 221]. Melanin nanoparticles effectively alleviated sepsis-induced myocardial damage and improved cardiomyocyte function by inhibiting ROS production and blocking the ferroptosis pathway to minimize oxidative stress, attenuate inflammation, and maintain mitochondrial balance [220]. In addition, in a model of ischemia-reperfusion renal injury, polydopamine nanoparticles loaded with rutin (PPR-NPs) showed protection against renal injury by scavenging ROS, protecting mitochondria, and inhibiting ferroptosis [221]. Notably, a recent comprehensive review summarized advancements in nanotechnology-based modulation of sepsis, with particular focus on oxidative stress and inflammation, 2 critical components of ferroptosis-associated injury, in an attempt to clarify the mechanistic roles of nanoparticles in modulating ferroptosis, which may include: 1) enhanced delivery of ferroptosis inhibitors such as Fer-1 and Lip-1 via functionalized nanocarriers, improving bioavailability and organ-targeting efficiency; 2) preferential accumulation of nanoparticles in inflamed tissues through either enhanced permeability and retention effect or ligand-mediated targeting of inflamed endothelium; and 3) intrinsic ROS-scavenging and iron-chelating properties of certain nanomaterials, such as melanin-like or catechol-based nanoparticles, which mitigate lipid peroxidation and labile iron overload-key drivers of ferroptosis [222]. These multifunctional features collectively position nanoparticles as versatile and powerful tools for regulating ferroptosis in sepsis settings. Other studies have explored the role of nanoparticles in the management of bacterial infections, particularly in inhibiting bacterial proliferation through ferroptosis-like mechanisms. Mesoporous calcium silicate nanoparticles trigger the classic ferroptosis pathway by increasing the levels of ROS and lipid peroxides, while reducing the GSH level in bacteria, whereas iron-loaded lipid nanoparticles can induce ferroptosis-like cell death in a wound infection model, thereby inhibiting bacterial proliferation, suggesting that the potential application of nanoparticles in sepsis is not limited to the regulation of ferroptosis but also includes antibacterial effects [223, 224]. These findings indicate that nanoparticle-mediated interventions can effectively regulate ferroptosis, with promise in controlling bacterial infection in the early stage of sepsis and mitigating sepsis-induced organ damage.

### Targeting ferroptosis to prevent the transition of sepsis from the hyperinflammatory phase to the immunosuppressive phase

The shift in sepsis from a hyperinflammatory state to an immunosuppressive phase marks a critical point in disease progression, often resulting in immune dysfunction and increased susceptibility to secondary infection [4]. Ferroptosis plays a central role in this transition by aggravating immune cell injury and dysfunction, thereby facilitating the switch from hyperinflammation to immunosuppression. Consequently, targeting ferroptosis has emerged as a potential therapeutic approach to preserve immune homeostasis in sepsis. Macrophages, which are essential regulators of both iron metabolism and inflammatory responses, are particularly vulnerable to ferroptosis. In sepsis, excessive iron accumulation in red pulp macrophages heightens their sensitivity to ferroptosis, a process that can be alleviated by the ferroptosis inhibitor Fer-1 [130]. By reducing oxidative stress and lipid peroxidation, ferroptosis inhibitors help preserve macrophage function. Furthermore, in the early stage of sepsis, macrophage polarization skews toward the M1 phenotype, amplifying inflammatory responses. Interventions at the level of ferroptosis, particularly in M2 macrophages, can mitigate this imbalance and prevent immune dysfunction. Lip-1, a commonly used inhibitor of lipid peroxidation and ferroptosis, has been shown to protect macrophages in this context [131]. In addition, during the hyperinflammatory phase, Fer-1 reduces neutrophil recruitment, thereby limiting excessive inflammation and tissue damage [141].

As sepsis advances into the immunosuppressive phase, ferroptosis further drives disease progression by intensifying immunosuppressive mechanisms, especially within M2 macrophages. DAMPs released by ferroptotic cells, such as HMGB1, reinforce immunosuppressive signals by promoting IL-10 production and downregulating MHC-II expression to impair the capacity of antigen presentation [161]. This impairment reduces the host’s ability to clear pathogens and increases the risk of secondary infections, a hallmark of late-stage sepsis. Thus, therapeutic strategies aimed at inhibiting ferroptosis in M2 macrophages may help sustain their function and counteract immunosuppression. Additionally, antioxidant supplementation, such as vitamin E or selenium, can restore GPX4 activity in T cells, protecting them from ferroptosis and preserving their immune function [142]. Enhancing GPX4 activity in both CD4^+^ and CD8^+^ T cells represents a promising strategy to prevent immunosuppression during the late stages of sepsis [143].

### Targeting ferroptosis to protect sepsis-associated multiple organ injury

Myocardial injury is a common feature of sepsis-induced MODS, often progressing to septic cardiomyopathy. In LPS-induced septic cardiomyopathy models, ferroptosis markers such as cyclooxygenase-2 and MDA are upregulated, leading to mitochondrial dysfunction in cardiomyocytes, accelerated cell death, and ultimately heart failure [125, 193]. Ferroptosis inhibition shows therapeutic promise, such as Fer-1 reduces lipid peroxides, protects cardiomyocytes and other cells, and improves cardiac function [125, 195], while Dex suppresses oxidative stress and iron overload in cardiomyocytes by upregulating GPX4 expression [196]. Beyond the heart, pharmacological interventions targeting ferroptosis also show therapeutic potential in SA-AKI. Agents such as ginsenoside Rg1 and melatonin protect renal tissues by regulating iron metabolism and enhancing GPX4 activity via the NRF2/HO-1 pathway, thereby reducing lipid peroxidation and ferroptosis [178, 179]. Similarly, Fer-1 and Lip-1 mitigate kidney injury by decreasing mitochondrial ROS generation [121, 180]. Likewise, in SA-ALI, ferroptosis drives alveolar epithelial cell death and respiratory failure. In LPS-induced ALI models, there were significantly downregulated GPX4 and SLC7A11 with substantially elevated MDA and ROS, while drugs like UA and uridine alleviated lung damage by activating NRF2 signaling, upregulating GPX4 and SLC7A11 expression, and inhibiting lipid peroxidation [174, 175]. Moreover, acupuncture protects alveolar epithelial cells via alpha 7 nicotinic acetylcholine receptor activation, further confirming the role of ferroptosis in SA-ALI [225]. In addition, ferroptosis contributes to SA-AHI and septic encephalopathy. By upregulating GPX4 expression, irisin alleviates sepsis-induced liver damage and septic encephalopathy [123, 200], while transferrin prevents ferroptosis by promoting iron sequestration through the SLC39A14-mediated non-transferrin-bound iron uptake pathway [226]. Taken together, ferroptosis plays a central role in sepsis-associated multiple organ damage. Targeting ferroptosis with agents such as Fer-1, Lip-1, irisin, and NRF2 activators may effectively preserve brain, heart, kidney, lung, and liver function. Further research and clinical application of these strategies may improve outcomes in patients with sepsis-related organ failure.

### Potential obstacles in translating ferroptosis-based therapies into the clinical setting

Mounting evidence has demonstrated that targeting ferroptosis with iron chelators, RTAs, GSH-GPX4 activators, and nanoparticle-based interference effectively prevents the pathological progression of sepsis by ameliorating sepsis-initiated immunosuppression and alleviating sepsis-associated multi-organ damage in animal and preclinical studies (Table 1) [120, 125, 128, 131, 144, 178, 181, 195, 196, 202, 206–210, 212]. Nevertheless, these encouraging protective effects on sepsis afforded by targeting ferroptosis hold great promise for translating ferroptosis-based therapies into the clinical setting. Yet, many ferroptosis-targeting compounds with promising therapeutic efficiency in both the cell system and animal models exhibit low solubility, high metabolic clearance, and low cellular permeability. In addition, some compounds display severe side effects and systemic drug toxicity, thus precluding them from clinical development and translation. For instance, Fer-1, as a potent RTA, confers protection against SA-ALI, SA-AKI, septic cardiomyopathy, septic encephalopathy, and sepsis-initiated immunosuppression [130, 180, 195, 202]; however, the metabolic instability and poor pharmacokinetics of Fer-1 make it unsuited for clinical drug development [227]. Another RTA, Lip-1, which has therapeutic effects on sepsis similar to Fer-1, strongly inhibits cytochrome P450 2D6 (CYP2D6), a key enzyme for drug metabolism and elimination, suggesting that Lip-1 may also be unsuitable as a clinical therapeutic agent [121, 131, 180, 195, 227]. On the other hand, iron chelators such as DFO have been reported to cause toxic side effects, including hearing loss and retinal abnormalities [228]. By boosting GSH synthesis and GPX activity, N-acetylcysteine improves outcomes in septic mice [206, 207], but its poor bioavailability necessitates high doses and prolonged treatment, raising the risk of anaphylactoid reaction, fluid overload, and high fluid osmolarity [229]. Of note, although the application of various iron chelators proves to be beneficial for protecting against sepsis-associated multi-organ damage [125, 208–210], some pathogens can exploit specific mechanisms to acquire iron from iron chelators such as DFO to support their growth and reproduction [230]. Therefore, to overcome these obstacles in translating ferroptosis-based therapeutic strategies into the clinical setting, a great effort is needed to develop more effective and highly selective ferroptosis-targeting compounds with better water solubility and cellular permeability, better metabolic stability and pharmacokinetics, and less or even neglected systemic toxicity.

Despite the promising therapeutic potential of nanoparticle-based ferroptosis inducers, several delivery-related challenges hinder their clinical translation. Toxicity remains a major concern, as iron-based nanoparticles can induce excessive oxidative stress, inflammation, and organ accumulation, particularly in the liver and kidneys, potentially exacerbating sepsis-induced multiorgan failure [220, 231]. Biodistribution issues, including poor tumor or infection site penetration due to size, charge, and physiological barriers, further limit the efficacy, while instability in biological fluids and low targeting specificity contribute to off-target effects and reduced therapeutic potency [232, 233]. Strategies such as PEGylation can reduce immune recognition but may also compromise cellular uptake and therapeutic efficacy [234]. Building on preclinical insights, recent studies have begun to explore the translational relevance of ferroptosis-related biomarkers in clinical settings. While emerging clinical evidence supports the feasibility of detecting biomarkers, including GPX4, ACSL4, and MDA, for monitoring ferroptosis in human sepsis, validation in large human cohorts is needed to establish clinical utility [76, 235]. Moreover, the interventional clinical trials that directly target ferroptosis pathways in sepsis patients remain lacking.

## Conclusion and perspectives

This review article systematically explores the role of ferroptosis in the pathological development and progression of sepsis, revealing how ferroptosis exacerbates the course of sepsis while promoting sepsis-related multi-organ damage and functional failure through mechanisms such as lipid peroxidation, iron metabolism disorders, as well as GSH depletion. As research progresses, the role of ferroptosis as a unique form of programmed cell death in sepsis is gradually being uncovered, offering a perspective on the pathological mechanisms of sepsis and providing potential targets for the innovation of clinical therapeutic strategies. The main innovations of this review are primarily reflected in the following aspects: 1) proposing a novel framework for the “biphasic immune regulation” of ferroptosis in the progression of sepsis; 2) systematically integrating the “ferroptosis-inflammation-immunity-multi-organ damage” network; 3) in-depth analysis of sepsis-specific ferroptosis regulatory networks; 4) expanding therapeutic strategies from single-target to comprehensive interventions; and 5) analyzing translational bottlenecks and optimization directions from a clinical perspective.

Although the role of ferroptosis in sepsis has garnered widespread attention, many unresolved issues remain. Firstly, the specific molecular regulatory networks of ferroptosis and its interactions with other forms of cell death, such as apoptosis and pyroptosis, are not yet fully understood. Secondly, most research remains confined to animal and cell models, and no interventional clinical trials have yet directly targeted ferroptosis pathways in sepsis patients, which means that translating these research findings into clinical applications faces multiple challenges, including drug delivery efficiency and safety. Thirdly, the etiology of sepsis is complex, and the physiological status and disease progression of patients exhibit high heterogeneity. Future studies should clarify patients’ specific pathological characteristics and optimize treatment through precision medicine. Finally, given the complexity of sepsis, single-target therapy often fails to achieve ideal outcomes. Combining ferroptosis inhibitors with other anti-inflammatory and immunomodulatory drugs may enhance the therapeutic effect and reduce sepsis-associated organ damage.

## Data Availability

Not applicable.

## Abbreviations

4-HNE: 4-Hydroxynonenal
ACSL: Acyl-CoA synthetase long-chain family member
AHI: Acute hepatic injury
AKI: Acute kidney injury
ALI: Acute lung injury
BH2: Dihydrobiopterin
BH4: Tetrahydrobiopterin
ceRNA: Competing endogenous RNA
CLP: Cecal ligation and puncture
CoQ10: Coenzyme Q10
cPLA2: Cytosolic phospholipase A2
DAMPs: Damage-associated molecular patterns
DCs: Dendritic cells
Dex: Dexmedetomidine
DFO: Deferoxamine
DHODH: Dihydroorotate dehydrogenase
DPP4: Dipeptidyl peptidase 4
DUSP1: Dual specificity protein phosphatase 1
ERK: Extracellular signal-regulated kinase
Fer-1: Ferrostatin-1
PGE2: Prostaglandin E2
FPN1: Ferroportin-1
FSP1: Ferroptosis suppressor protein 1
GCH1: GTP cyclohydrolase 1
GOT1: Glutamic-oxaloacetic transaminase 1
GPX4: Glutathione peroxidase 4
GSH: Glutathione
HLA-DR: Human leukocyte antigen-DR isotype
HMGB1: High mobility group box 1
HO-1: Heme oxygenase-1
ICU: Intensive care unit
IL: Interleukin
IL4i1: Interleukin-4 induced-1
IRP: Iron regulatory protein
LBP: Lipopolysaccharide-binding protein
Lcn2: Lipocalin-2
Lip-1: Liproxstatin-1
LncRNA: Long non-coding RNA
LOOH: Lipid hydroperoxide
LPCAT3: Lysophosphatidylcholine acyltransferase 3
LPS: Lipopolysaccharide
MAPK: Mitogen-activated protein kinase
MDA: Malondialdehyde
MDSCs: Myeloid-derived suppressor cells
MHC: Major histocompatibility complex
MODS: Multiple organ dysfunction syndrome
NADPH: Nicotinamide adenine dinucleotide phosphate
NCF2: Neutrophil cytosol factor 2
NCOA4: Nuclear receptor coactivator 4
NEAT1: Nuclear enriched abundant transcript 1
NEDD4L: Neural precursor cell expressed developmentally downregulated gene 4-like
NETs: Neutrophil extracellular traps
NK: Natural killer
NLRP3: NLR family pyrin domain-containing 3
NRAMP2: Natural resistance-associated macrophage protein 2
NRF2: Nuclear factor erythroid 2-related factor 2
OXPHOS: Oxidative phosphorylation
PEs: Phosphatidylethanolamines
PPARγ: Peroxisome proliferator-activated receptor gamma
PRRs: Pattern recognition receptors
PTGS2: Prostaglandin-endoperoxide synthase 2
PUFA: Polyunsaturated fatty acid
Rab26: Ras-associated protein 26
RCD: Regulated cell death
ROS: Reactive oxygen species
RSL3: RAS-selective lethal 3
RTA: Radical-trapping antioxidant
SA-AII: Sepsis-associated acute intestinal injury
SA-AKI: Sepsis-associated acute kidney injury
SA-ALI: Sepsis-associated acute lung injury
SA-E: Sepsis-associated encephalopathy
SLC7A11: Solute carrier family 7 member 11
SOFA: Sequential organ failure assessment
SQSTM1: Sequestosome 1
STEAP3: Six-transmembrane epithelial antigen prostate 3
TFR1: Transferrin receptor 1
TFRC: Transferrin receptor
Tfh: Follicular helper T
TGF-β: Transforming growth factor-β
TNF-α: Tumor necrosis factor-α
Tregs: Regulatory T cells
UA: Urolithin A
YAP1: Yes-associated protein 1
